# Knowledge, practice and attitude toward anabolic hormones and nutritional supplements among people practicing sports in the MENA region before and during COVID-19 lockdown

**DOI:** 10.3389/fpubh.2022.1018757

**Published:** 2022-10-17

**Authors:** Nael Kamel Eltewacy, Soha Nabil, Rahma Sweedy, Roy Rillera Marzo, Nouran Hamza, The EARG Group

**Affiliations:** ^1^Faculty of Pharmacy, Beni-Suef University, Minia, Egypt; ^2^Faculty of Medicine, Beni-Suef University, Beni Suef, Egypt; ^3^Medical Agency for Research and Statistics (MARS), Cairo, Egypt; ^4^Department of Community Medicine, International Medical School, Management and Science University, Shah Alam, Selangor, Malaysia; ^5^Global Public Health, Jeffrey Cheah School of Medicine and Health Sciences, Monash University, Subang Jaya, Selangor, Malaysia; ^6^Clinical Research Key (CRK-CRO), Nairobi, Kenya

**Keywords:** coronavirus, knowledge, attitude, practice, supplements, hormones, sports, MENA

## Abstract

**Introduction:**

During the COVID-19 lockdown, people's lifestyles have changed including their habits and physical activities. There has been an increase in anabolic hormones and nutritional supplement use among people who regularly do exercise in the MENA region. This study aims to assess knowledge, practice, and attitude toward the use of anabolic hormones and nutritional supplements among people who regularly exercise in the Middle East and North Africa (MENA) region and to compare their exercise habits and hormones and supplements usage between before and during COVID-19 lockdown.

**Methods:**

A self-administrated online Google form survey was carried out between February 2021 and April 2021. Five thousand eight hundred forty-five participants who regularly exercise and aged ≥18 years responded to the questionnaire. The questionnaire was distributed through social media platforms and included five sections: demographic, training characters, knowledge, practice, and attitude.

**Results:**

The participants mean age was 27.4 ± 8.6 years. Males represented 58.2 % of participants. 75.3% of the study participants had not used either hormones or supplements, and about 19% used supplements only. The mean percent score for knowledge, practice, and attitude were 39.3 ± 30.5, 1.1 ± 9.5, and 21.3 ± 23.8, respectively. Level of knowledge was higher among participants who worked in the medical field or as sports coaches. The practice was higher among male participants. The most commonly used anabolic hormones and nutritional supplements were steroids and proteins with bodybuilding being the most common purpose. Internet was the main source of information and pharmacy was the main source for procuring these substances. There was a significant decrease in proteins, carbohydrates, and sports drinks used during the COVID-19 lockdown compared to before the COVID-19 lockdown, while a statistically significant increase in vitamins used during the COVID-19 lockdown compared to before COVID-19 lockdown.

**Discussion:**

In the MENA region, there has been an increase in the use of anabolic hormones and nutritional supplements. Most of the population has low knowledge of the harmful effect of uncontrolled, uninformed and unmonitored use of these substances Therefore, increasing the awareness level of participants and sports coaches should be a priority to limit the unsupervised use of hormones and supplements.

## Introduction

In January 2020, World Health Organization (WHO) proclaimed coronavirus disease 2019 (COVID-19) to be a public health emergency and identified the disease as a global pandemic on 11 March 2020 (1, 2). COVID-19 is an extremely infectious disease caused by a virus called severe acute respiratory syndrome coronavirus-2 (SARS-CoV-2) that is a member of the coronaviruses family (3). This highly contagious virus was first reported in Wuhan city, China and then spread to affect over 1.1 million cases in the Eastern Mediterranean Region as reported by the WHO in July 2020 (4). Based on WHO recommendations, governments all over the world begun to enforce social distancing, quarantine, and isolation to limit the disease spread (4, 5). Countries in the Middle East and North Africa (MENA) region started to close schools, religious places, malls, airports, and some countries even suspended the government departments (6). During this tenure of social distance and lockdown, people's behaviors and lifestyles have changed and these changes include eating habits and physical activities (7). Personal restrictions also can result in a lack of physical activity, especially in countries with complete lockdown, such as Jordan (8).

Currently, anabolic hormones and nutritional supplements are used widely in people associated with sports. People, who exercise, use these substances for different reasons, for example, to improve their abilities, to strengthen their muscles, or to look better (9). Anabolic hormones, such as insulin and testosterone, are substances that help in cellular growth by triggering the anabolic pathway. These substances can be also developed in labs as is the case with anabolic steroids (10). While nutritional supplements are concentrated forms of food components, vitamins and minerals are used mainly to improve health and avoid diseases (11, 12).

In the Middle East, there has been an increase in consumption of anabolic hormones and nutritional supplements. In Saudi Arabia, a study reported that among gym attendants around 7.9% use anabolic hormones, and 47.9% use nutritional supplements (9). While the percent of hormone users was 22.7% in Kuwait (13) and 22% in United Arab Emirates (UAE) (14). Studies reported that a large number of individuals who use these substances did not consult doctors before taking them (15, 16). Sports authorities and the public in Arab countries started to pay more attention to the effect of using these substance on the wellbeing of healthy young people (17). Inappropriate use of hormones and supplements may lead to serious side effects. In the case of hormones, it may lead to high blood pressure, infertility, prostate cancer, increased libido, mood swings, and aggressiveness (18, 19), while in the case of supplements, it may cause cardiovascular problems, kidney failures, and fluid retention (20).

There is limited information regarding people's knowledge, practice, and attitude toward the use of anabolic hormones and nutritional supplements in MENA region and if there was any change in their habits during COVID-19 lockdown. Therefore, this study aims to assess knowledge, practice and, attitude toward the use of anabolic hormones and nutritional supplements among people who regularly exercise in MENA region and to compare their habits for using anabolic hormones, nutritional supplements and for exercising between before and during COVID-19 lockdown.

## Methodology

### Study design and participants

This multicenter cross-sectional study was conducted in MENA region through an online survey between February 2021 and April 2021. The study was carried out across eighteen countries (Algeria, Bahrain, Egypt, Iraq, Jordan, Kuwait, Lebanon, Libya, Morocco, Palestine, Qatar, Saudi Arabia, Sudan, Syria, UAE, and Yemen). Male and female participants with a minimum age of 18 years who regularly exercise were included in the study. We used convenience and snowball sampling method, in which we collected the data from participants who were accessible to fill the questionnaire. The sample size was calculated according to Charan and Biswas (21) and Arkin (22) equations with a minimum of 400 participants from each country (21, 22).

### Study survey

The questionnaire was a self-administrated online Google form survey, available in Arabic and English languages. The questionnaire was divided into 5 main domains including: (1) Socio-demographic characteristics: age, sex, country geographic location, educational level, monthly income, job, marital status, smoking, weight before and during COVID-19 lockdown, fat percentage before and during COVID-19 lockdown, and height; (2) Training characteristics: total period of exercise, exercise frequency, diet, going to the gym; (3) Knowledge about hormones and supplements: side effects of anabolic hormones, nutritional supplements side effect, source of their information, who advised them to use it; (4) Practice regarding hormones and supplements use: types of hormones used, route of administration for hormones, types of supplements used, following with nutritionist, source for buying these substances, withdrawal symptoms if they stopped; (5) Attitude toward hormones and supplements: opinions regarding hormones and supplements benefits, reasons for the use, attitude toward the use of these substances.

### Validation and pilot study

The questionnaire was designed based on previous studies in Saudi, Emirates, and Kuwait (9, 13, 14, 23). With an aim to validate the survey, five experts from the field of nutrition were requested to fill the online Google form survey. These experts evaluated the degree of relevance of each question in the questionnaire and if it can correctly measure knowledge, practice, and attitude toward the use of anabolic hormones and nutritional supplements. Post validation, pilot study was conducted in 20 to 30 participants from 16 different countries in the MENA region. Their reliability and internal consistency of the survey was assessed using Cronbach's alpha which was 0.911 for knowledge section and 0.769 for the attitude section.

### Data collection

On the first page of the Google form, an option was provided to use one of two languages (Arabic or English). This helped participants from countries whose main language is not only Arabic, for example Morocco and Iraq, to participate in the study. An online link was distributed through different social media platforms. People who agreed to participate used the link to access the survey that did not collect any personal or contact details.

### Ethical consideration

Ethical approval was obtained from Institutional review board Committee (IRB) at the Sahel General Hospital, Lebanon. Participant's anonymity and confidentiality was ensured throughout the study and analysis. If participants submitted the answered survey, we considered that as consent to participate in the study.

### Statistical analysis

Data from the online questionnaire was collected, verified, and used for statistical analysis using R Software version 3.5.2 (2018-12-20) – Eggshell Igloo. For baseline demographic and training characteristics, mean and standard deviation were used for continuous data, and count and percent were used for categorical data. A score of 0 and 2 was assigned for the answers in each knowledge, practice, and attitude section, where 0 represented the worst and 2 the best. Regarding questions of scaled answers, all answers below neutral were assigned a score of 0 and all answers above neutral were scored as 2 for easier scoring scale. For some KAP responses, at which scores were not be applicable, count and percent were used for description after excluding minor and inconsistent responses. A spearman correlation was analyzed between each two domains as the distribution of total scores of each of knowledge, attitude and practice had violated the normal assumption.

## Results

From 5,845 responders to the questionnaire, 5,353 subjects (91.6%) were completely responding. The **knowledge** domain consists of 21 questions with 48.2% complete responding, the **attitude** domain consists of 7 questions with 48.5% complete responding and **practice** domain consists of 10 questions with 48.5% complete responding (Among those who were consuming hormones or supplements or both were). Inconsistent responds were excluded from the analysis which were 5 responds (0.1%) related to (age), 325 responds (5.6%) related to [Since when you started to do exercise? (months)], 89 responds (1.5%) related to (Your monthly income in dollar), 80 responds(1.4%) related to Fat percentage during COVID-19 lockdown, and 63 responds (1.1%) related to Fat percentage before COVID-19 lockdown.

A total of 5,845 participants responded to the questionnaire with a mean age of 27.4 ± 8.6 years. More than half of the participants were male (58.2%) and most of the participants were urban (85.7%). In terms of educational qualification, 61.5% of participants had Bachelors, Masters or a Doctorate degree and 19.8% had secondary, intermediate, or higher secondary education. Professionally, regarding participants' occupations, it was observed that 27.6% of the study participants were working in the medical field while only 11.3% of participants were sports coaches; 39.9, 28.2, and 19.5% of participants were students, worked at private sector, and worked at Government sector, respectively. 70.3% of participants were single, 27.5% were married, 1.6% were divorced and 0.6% were widowed. The average monthly income of the participants was 740.0 ± 1474.6 US dollars. The average weight of the participants before and during COVID-19 lockdown was 73.0 ± 18.2 and 74.1 ± 21.1 kg, respectively. The average height was 168.3 ± 17.6 cm. The average fat percentage before and after COVID-19 lockdown was 19.1 ± 7.4% and 20.0 ± 7.8%, respectively. Of all the participants, 77.6% were non-smokers. As shown in Table 1, 75.3% of the study population did not use either anabolic hormones or nutritional supplements, 19.8% used only the nutritional supplements, 0.8% used only the anabolic hormones and 4.1% used anabolic hormones and nutritional supplements (Table 1).

**Table 1:** Baseline demographic characteristics among study population.

**Demographics**	**Subgroups**	**Total (*N* = 5,845)**
Age	Mean (SD)	27.4 (8.6)
Sex	Female	2,442 (41.8)
	Male	3,403 (58.2)
Country of	Algeria	453 (7.8)
residence	Bahrain	179 (3.1)
	Egypt	539 (9.2)
	Iraq	424 (7.3)
	Jordan	375 (6.4)
	Qatar	66 (1.1)
	Kuwait	431 (7.4)
	Lebanon	449 (7.7)
	Libya	152 (2.6)
	Morocco	564 (9.6)
	Oman	1 (0.0)
	Palestine	438 (7.5)
	Saudi	438 (7.5)
	Sudan	445 (7.6)
	Syria	412 (7.0)
	Tunisia	3 (0.1)
	UAE	169 (2.9)
	Yemen	307 (5.3)
Geographic	Rural	836 (14.3)
location	Urban	5,009 (85.7)
Your highest	Bachelors/Masters/Doctorate	3,597 (61.5)
educational level	Diploma/Trade Qualification	1,026 (17.6)
	Primary	66 (1.1)
	Secondary/Intermediate/Higher Secondary	1,156 (19.8)
Do you work in the	No	4,230 (72.4)
medical field	Yes	1,615 (27.6)
Are you a sports	No	5,182 (88.7)
coach	Yes	663 (11.3)
Where do you work	Government Sector	1,138 (19.5)
	Housewife	234 (4.0)
	Private Sector	1,648 (28.2)
	Student	2334 (39.9)
	Unemployed	491 (8.4)
Marital state	Divorced	95 (1.6)
	Married	1,606 (27.5)
	Single	4,108 (70.3)
	Widowed	36 (0.6)
Your monthly income in dollar	Mean (SD)	740.0 (1,474.6)
Weight. Before. COVID-19 lockdown, Kg	Mean (SD)	73.0 (18.2)
Weight. during. COVID-19 lockdown, Kg	Mean (SD)	74.1 (21.1)
Height, cm	Mean (SD)	168.3 (17.6)
Fat percentage before COVID-19 lockdown	Mean (SD)	19.1 (7.4)
Fat percentage during COVID-19 lockdown	Mean (SD)	20.0 (7.8)
Do you smoke?	No	4,533 (77.6)
	Yes	1,312 (22.4)
Do you use hormones and supplements?	Both of them	240 (4.1)
	Hormones only	45 (0.8)
	None-of them	4,404 (75.3)
	Supplements only	1,156 (19.8)

Regarding training characteristics of the participants, 45.9% of the study population used to go to the gym before COVID-19 lockdown and 25.6% had a gym qualified trainer. The average duration since they started to do exercise was 18.3 ± 25.9 months. The frequency and duration of exercise by participants before and during COVID-19 lockdown are summarized in Table 2. Before COVID-19 lockdown, 30.1% of the study population was following a special diet while 30.6% of participants followed a special diet during COVID-19 lockdown (Table 2).

**Table 2:** Baseline training characteristics among study population.

**Training characters**	**Subgroups**	**Total (*N* = 5,845)**
Since when started to do exercise (months)	Mean (SD)	18.3 (25.9)
How many times you exercise per week before COVID-19 lockdown?	Five times or more per week	988 (16.9)
	Four times a week	932 (15.9)
	Once a week	1,915 (32.8)
	Three times a week	1,135 (19.4)
	Twice a week	875 (15.0)
How many times you exercise per week during COVID-19 lockdown?	Five times or more per week	784 (13.4)
	Four times a week	773 (13.2)
	Once a week	2,307 (39.5)
	Three times a week	992 (17.0)
	Twice	989 (16.9)
How many hours you exercise per day before COVID-19 lockdown?	From half an hour to 1 h	1,856 (31.8)
	From 1 to 2 h	1,807 (30.9)
	Less than half an hour	1,811 (31.0)
	More than 2 h	371 (6.3)
How many hours you exercise per day during COVID-19 lockdown?	From half an hour to 1	2,073 (35.5)
	From 1 to 2 h	1,464 (25.0)
	Less than half an hour	2,046 (35.0)
	More than 2 h	262 (4.5)
Do you follow a special diet before COVID-19 lockdown?	No	4,088 (69.9)
	Yes	1,757 (30.1)
Do you follow a special diet during COVID-19 lockdown?	No	4,055 (69.4)
	Yes	1,790 (30.6)
Do you go to the gym?	No	3,165 (54.1)
	Yes	2,680 (45.9)
Is the trainer in the gym qualified has a certificate?	I don't go to the gym	2,381 (40.7)
	I don't know	1,233 (21.1)
	No trainer in the gym	291 (5.0)
	Not qualified	444 (7.6)
	Yes	1,496 (25.6)

**Figure 1:**
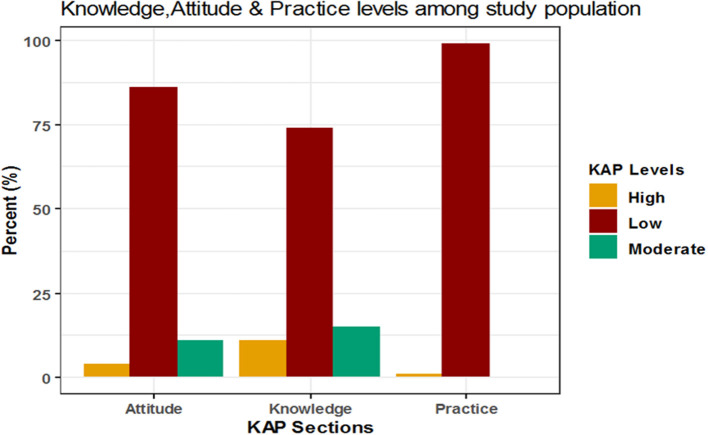
Levels of knowledge, attitude and practice.

**Figure 2:**
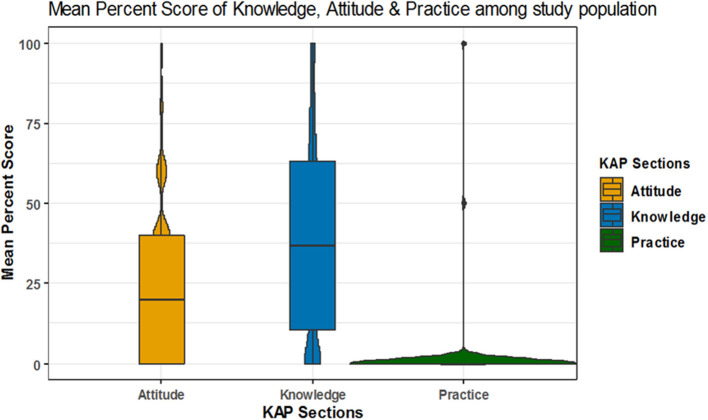
Mean percent score of knowledge, attitude and practice.

**Figure 3:**
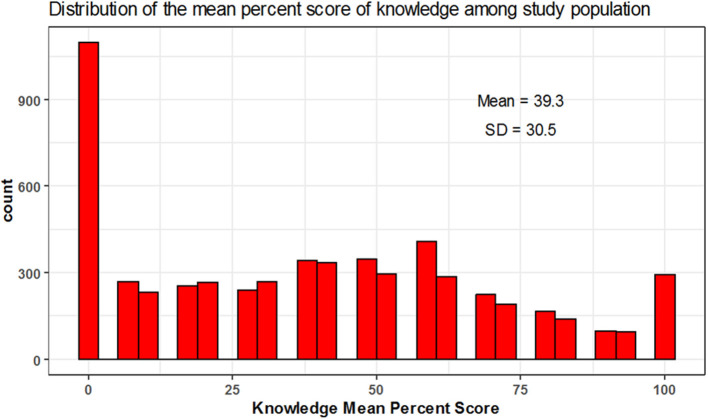
Mean percent score distribution for knowledge.

**Figure 4:**
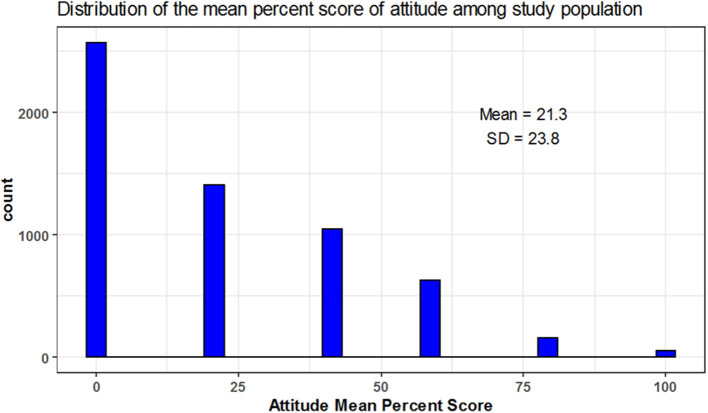
Mean percent score distribution for attitude figure.

**Figure 5:**
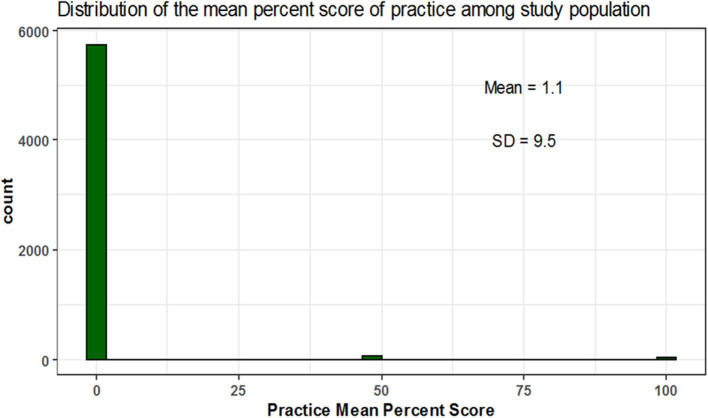
Mean percent score distribution for practice.

The mean percent score of knowledge was 39.3 ± 30.5 and 11, 15, and 74% of participants were of high, moderate and low knowledge level, respectively. The mean percent score for attitude was 21.3 ± 23.8 with 4, 11, and 86% of participants had high, moderate, low attitude level, respectively. The mean percent score for practice was 1.1 ± 9.5; 1% of the participants showed high practice level while the remaining (99%) showed low practice level and none of the participants showed a moderate level in practice (Table 3 and Figures 1–5).

**Table 3:** The mean percent score for knowledge, attitude and practice.

		**Knowledge**	**Attitude**	**Practice**
Mean percent score	Mean ± SD	39.3 ± 30.5	21.3 ± 23.8	1.1 ± 9.5
KAP levels	High level	628 (11%)	205 (4%)	40 (1%)
	Moderate level	866 (15%)	622 (11%)	0 (0%)
	Low level	4,351 (74%)	5,018 (86%)	5,805 (99%)

**Figure 6:**
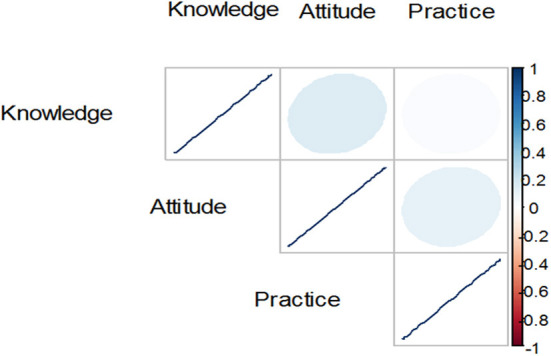
Correlation between knowledge, attitude, and practice.

As shown in Table 4 and Figure 6, knowledge level was positively correlated with attitude level with a very weak association (r = 0.14) and also positively correlated with practice level with a very weak association that can be negligible (r = 0.02) while the attitude level was also positively correlated with the practice level with a very weak association (r = 0.10).

**Table 4:** Correlation between knowledge, attitude and practice total score.

	**Knowledge**	**Attitude**	**Practice**
Knowledge	1.00	0.14	0.02
Attitude	0.14	1.00	0.10
Practice	0.02	0.10	1.00

### Univariate logistic regression model for the association between demographics and high level of knowledge

For each one-year increase in participant's age the odds of high level of knowledge increased significantly by 1% (OR = 1.01, 95% CI: 1.00–1.02, *p* = 0.031). Also Egyptian, Jordanian, Qatari, Kuwaiti, Lebanese, and Emirati participants showed significantly increased odds of high level of knowledge by 2.18 folds (OR = 2.18, 95% CI: 1.42–3.43, *p* = 0.001), 2.72 folds (OR = 2.72, 95% CI: 1.73–4.35, *p* < 0.001), 4.41 folds (OR = 4.41, 95% CI: 2.25–8.47, *p* < 0.001), 2.33 folds (OR = 2.33, 95% CI: 1.50–3.69, *p* < 0.001), 2.16 folds (OR = 2.16, 95% CI: 1.38–3.43, *p* = 0.001) and 2.19 folds (OR = 2.19, 95% CI: 1.25–3.82, *p* = 0.006) folds, respectively. On the contrary, Yemeni participants showed significantly decreased odds of high knowledge level by about 53% when compared to Algerian participants (OR = 0.47, 95% CI: 0.23–0.90, *p* = 0.029).

Some difference in knowledge level was observed based on the qualification of the participants. Participants with diploma/trade qualification, primary and secondary, intermediate, higher secondary education showed significantly decreased odds of high knowledge level by nearly 37% (OR = 0.63, 95% CI: 0.49–0.80, *p* < 0.001), 81% (OR = 0.19, 95% CI: 0.03–0.60, *p* = 0.019) and 48% (OR = 0.52, 95% CI: 0.41–0.66, *p* < 0.001), respectively, when compared to participants with bachelors, masters, or doctorate degree.

Based on the occupation, it was found that the participants working in the medical field showed significantly increased odds of high knowledge level by 2.39 folds when compared to participants who do not work in the medical field (OR = 2.39, 95% CI: 2.01–2.84, *p* < 0.001). Also, sports coaches showed significantly increased odds of high knowledge level by 51% when compared to participants who are not sport coached (OR = 1.51, 95% CI: 1.17–1.91, *p* = 0.001). Also, participants working in private sector, studying and unemployed participants showed significantly decreased odds of high knowledge level by 33% (OR = 0.67, 95% CI: 0.53–0.85, *p* = 0.001), 24% (OR = 0.76, 95% CI: 0.62–0.95, *p* = 0.016), and 48% (OR = 0.52, 95% CI: 0.36–0.75, *p* = 0.001), respectively, when compared to participants working in government sector.

Fat percentage also correlated with the level of knowledge. The odds of high knowledge level increased significantly by 2% (OR = 1.02, 95% CI: 1.00–1.03, *p* = 0.006) and 1% (OR = 1.01, 95% CI: (1.00–1.02), *p* = 0.013) for each one unit increase in the fat percentage in participants before COVID-19 lockdown and during COVID-19 lockdown, respectively.

Table 5 shows that the odds of high knowledge level also increased significantly by 2.81 folds (OR = 2.81, 95% CI: 1.01–7.24, *p* = 0.037) and 99% (OR = 1.99, 95% CI: 1.21–3.54, *p* = 0.012) among participants who used anabolic hormones only and among participants who did not use either anabolic hormones or nutritional supplements, respectively, when compared to the participants who used both the anabolic hormones and nutritional supplements.

**Table 5:** Logistic regression models for the association between demographics and high level of knowledge.

**Demographics**		**Low**	**High**	**OR (univariable)**	**OR (multivariable)**
Age	Mean (SD)	27.3 (8.7)	28.1 (8.9)	1.01 (1.00–1.02, *p* = 0.031)	1.01 (1.00–1.03, *p* = 0.170)
Sex	Female	1,789 (86.6)	276 (13.4)	–	–
	Male	2,562 (87.9)	352 (12.1)	0.89 (0.75–1.05, *p* = 0.178)	1.07 (0.85–1.36, *p* = 0.551)
Country of residence	Algeria	337 (91.3)	32 (8.7)	–	–
	Bahrain	136 (87.2)	20 (12.8)	1.55 (0.84–2.78, *p* = 0.148)	2.10 (1.11–3.90, *p* = 0.021)
	Egypt	357 (82.8)	74 (17.2)	2.18 (1.42–3.43, *p* = 0.001)	1.82 (1.15–2.95, *p* = 0.012)
	Iraq	331 (91.4)	31 (8.6)	0.99 (0.59–1.66, *p* = 0.958)	1.11 (0.64–1.94, *p* = 0.711)
	Jordan	236 (79.5)	61 (20.5)	2.72 (1.73–4.35, *p* < 0.001)	2.45 (1.51–4.05, *p* < 0.001)
	Qatar	43 (70.5)	18 (29.5)	4.41 (2.25–8.47, *p* < 0.001)	6.03 (2.90–12.39, *p* < 0.001)
	Kuwait	303 (81.9)	67 (18.1)	2.33 (1.50–3.69, *p* < 0.001)	2.50 (1.51–4.22, *p* < 0.001)
	Lebanon	307 (83.0)	63 (17.0)	2.16 (1.38–3.43, *p* = 0.001)	2.58 (1.60–4.24, *p* < 0.001)
	Libya	116 (91.3)	11 (8.7)	1.00 (0.47–1.99, *p* = 0.997)	1.13 (0.52–2.31, *p* = 0.752)
	Morocco	431 (88.0)	59 (12.0)	1.44 (0.92–2.29, *p* = 0.114)	1.74 (1.08–2.86, *p* = 0.025)
	Oman	1 (100.0)	0 (0.0)	0.00 (NA-Inf, *p* = 0.983)	–
	Palestine	337 (87.5)	48 (12.5)	1.50 (0.94–2.42, *p* = 0.092)	1.43 (0.87–2.38, *p* = 0.163)
	Saudi	347 (89.9)	39 (10.1)	1.18 (0.73–1.94, *p* = 0.501)	1.20 (0.69–2.11, *p* = 0.513)
	Sudan	377 (93.8)	25 (6.2)	0.70 (0.40–1.20, *p* = 0.195)	0.69 (0.39–1.22, *p* = 0.201)
	Syria	294 (87.5)	42 (12.5)	1.50 (0.93–2.46, *p* = 0.099)	1.44 (0.87–2.43, *p* = 0.161)
	Tunisia	3 (100.0)	0 (0.0)	0.00 (NA-Inf, *p* = 0.971)	0.00 (NA-Inf, *p* = 0.971)
	UAE	125 (82.8)	26 (17.2)	2.19 (1.25–3.82, *p* = 0.006)	2.64 (1.40–4.95, *p* = 0.002)
	Yemen	270 (95.7)	12 (4.3)	0.47 (0.23–0.90, *p* = 0.029)	0.42 (0.20–0.84, *p* = 0.018)
Geographic location	Rural	634 (87.4)	91 (12.6)	–	–
	Urban	3,717 (87.4)	537 (12.6)	1.01 (0.80–1.28, *p* = 0.957)	1.04 (0.81–1.35, *p* = 0.756)
Your highest educational level	Bachelors/Masters/Doctorate	2,564 (85.0)	452 (15.0)	–	–
	Diploma/Trade Qualification	793 (90.0)	88 (10.0)	0.63 (0.49–0.80, *p* < 0.001)	0.70 (0.54–0.90, *p* = 0.006)
	Primary	61 (96.8)	2 (3.2)	0.19 (0.03–0.60, *p* = 0.019)	0.11 (0.01–0.54, *p* = 0.034)
	Secondary/Intermediate/ Higher Secondary	933 (91.6)	86 (8.4)	0.52 (0.41–0.66, *p* < 0.001)	0.63 (0.48–0.82, *p* = 0.001)
Do you work in the medical field?	No	3,368 (90.1)	370 (9.9)	–	–
	Yes	983 (79.2)	258 (20.8)	2.39 (2.01–2.84, *p* < 0.001)	2.48 (2.03–3.02, *p* < 0.001)
Are you a sports coach?	No	3,916 (87.9)	538 (12.1)	–	–
	Yes	435 (82.9)	90 (17.1)	1.51 (1.17–1.91, *p* = 0.001)	2.10 (1.59–2.77, *p* < 0.001)
Where do you work?	Government Sector	808 (84.1)	153 (15.9)	–	–
	Housewife	174 (85.7)	29 (14.3)	0.88 (0.56–1.33, *p* = 0.560)	1.47 (0.89–2.38, *p* = 0.124)
	Private Sector	1,250 (88.8)	158 (11.2)	0.67 (0.53–0.85, *p* = 0.001)	0.75 (0.57–0.98, *p* = 0.038)
	Student	1,714 (87.4)	248 (12.6)	0.76 (0.62–0.95, *p* = 0.016)	1.11 (0.83–1.51, *p* = 0.478)
	Unemployed	405 (91.0)	40 (9.0)	0.52 (0.36–0.75, *p* = 0.001)	0.86 (0.57–1.29, *p* = 0.473)
Marital state	Divorced	71 (87.7)	10 (12.3)	–	–
	Married	1,196 (85.9)	197 (14.1)	1.17 (0.62–2.45, *p* = 0.651)	1.18 (0.59–2.65, *p* = 0.656)
	Single	3,054 (88.0)	417 (12.0)	0.97 (0.52–2.01, *p* = 0.928)	1.02 (0.50–2.32, *p* = 0.958)
	Widowed	30 (88.2)	4 (11.8)	0.95 (0.24–3.08, *p* = 0.931)	0.83 (0.17–3.21, *p* = 0.803)
Your monthly income in dollar	Mean (SD)	730.9 (1,473.2)	937.8 (1,720.2)	1.00 (1.00–1.00, *p* = 0.002)	1.00 (1.00–1.00, *p* = 0.940)
Weight before COVID-19 lockdown, kg	Mean (SD)	72.7 (17.9)	73.9 (16.7)	1.00 (1.00–1.01, *p* = 0.112)	0.99 (0.99–1.00, *p* = 0.190)
Weight during COVID-19 lockdown, kg	Mean (SD)	73.8 (20.9)	76.2 (26.2)	1.00 (1.00–1.01, *p* = 0.019)	1.01 (1.00–1.01, *p* = 0.043)
Height, cm.	Mean (SD)	168.0 (18.4)	168.5 (17.0)	1.00 (1.00–1.01, *p* = 0.584)	1.00 (0.99–1.01, *p* = 0.816)
Fat percentage before COVID-19 lockdown	Mean (SD)	19.0 (7.4)	19.9 (7.7)	1.02 (1.00–1.03, *p* = 0.006)	1.01 (0.99–1.04, *p* = 0.223)
Fat percentage during COVID−19 lockdown	Mean (SD)	19.9 (7.8)	20.7 (8.2)	1.01 (1.00–1.02, *p* = 0.013)	1.00 (0.98–1.02, *p* = 0.696)
Do you smoking?	No	3,361 (87.3)	490 (12.7)	–	–
	Yes	990 (87.8)	138 (12.2)	0.96 (0.78–1.17, *p* = 0.663)	1.01 (0.80–1.26, *p* = 0.962)
Do you use hormones and supplements?	Both of them	193 (92.8)	15 (7.2)	–	–
	Hormone only	32 (82.1)	7 (17.9)	2.81 (1.01–7.24, *p* = 0.037)	3.61 (1.22–9.98, *p* = 0.015)
	None of them	3,288 (86.6)	508 (13.4)	1.99 (1.21–3.54, *p* = 0.012)	1.84 (1.07–3.39, *p* = 0.038)
	Supplements only	838 (89.5)	98 (10.5)	1.50 (0.88–2.75, *p* = 0.157)	1.27 (0.72–2.40, *p* = 0.430)

### Adjusted logistic regression model for the association between demographics and high level of knowledge

The adjusted odds of high level of knowledge increased significantly among participants from Bahrain, Egypt, Jordan, Qatar, Kuwait, Lebanon, Morocco and UAE by about 2 folds (OR = 2.10, 95%CI: (1.11–3.90), *p* = 0.021), 1.8 folds (OR = 1.82, 95%CI: (1.15–2.95, *p* = 0.012), 2.4 folds (OR = 2.45, 95%CI: (1.51–4.05, *p* < 0.001), 6 folds) OR = 6.03, 95%CI: (2.90–12.39, *p* < 0.001), 2.5 folds (OR = 2.50, 95%CI: (1.51–4.22, *p* < 0.001), 2.6 folds (OR = 2.58, 95%CI: (1.60–4.24, *p* < 0.001), 74% (OR = 1.74, 95%CI: (1.08–2.86, *p* = 0.025) and 2.6 folds (OR = 2.64, 95%CI: (1.40–4.95, *p* = 0.002), respectively, when compared to Algerian participants. While the adjusted odds of high knowledge level decreased significantly among participants from Yemen by 58% when compared to Algerian participants (OR = 0.42, 95%CI: (0.20–0.84, *p* = 0.018).

Furthermore, the adjusted odds of high level of knowledge decreased significantly among participants with diploma, or /trade qualification, primary and secondary, intermediate, or /higher secondary education by 30% (OR = 0.70, 95%CI: (0.54–0.90, *p* = 0.006), 89% (OR = 0.11, 95%CI: (0.01–0.54, *p* = 0.034) and 37% (OR = 0.63 (0.48–0.82, *p* = 0.001), respectively, when compared to participants with bachelors, masters or doctorate degree. Also, the adjusted odds of high level of knowledge increased significantly among participants working in the medical field by about 2.5 folds (OR = 2.48, 95%CI: (2.03–3.02, *p* < 0.001) when compared to participants who did not. The adjusted odds of level of high level of knowledge also increased significantly among sport coaches by about 2 folds (OR = 2.10, 95%CI: (1.59–2.77, *p* < 0.001) when compared to other participants. The adjusted odds of high level of knowledge level decreased significantly among participants working in private sector by nearly 25% (OR = 0.75, 95%CI: (0.57–0.98, *p* = 0.038) when compared to participants working in government sector.

Next, we found that the adjusted odds of high level of knowledge increased significantly by about 1% (OR = 1.01, 95%CI: (1.00–1.01, *p* = 0.043) for one unit increase in the weight of participants during COVID-19 lockdown. It also increased significantly among participants who used only anabolic hormones only and participants who did not use either anabolic hormones or nutritional supplements by 3.6 folds (OR = 3.61, 95%CI: (1.22–9.98, *p* = 0.015) and 84% (OR = 1.84, 95%CI: (1.07–3.39, *p* = 0.038) respectively, when compared to participants who used both (Table 5).

### Univariate logistic regression model for the association between demographics and moderate level of knowledge

The odds of moderate level of knowledge decreased significantly among participants from Morocco, Palestine, Saudi, Sudan, UAE and Yemen by about 31% (OR = 0.69, 95% CI: 0.49–0.97, *p* = 0.033), 37% (OR = 0.63, 95% CI: 0.43–0.92, *p* = 0.016), 40% (OR = 0.60, 95% CI: 0.41–0.87, *p* = 0.008), 54% (OR = 0.46, 95% CI: 0.31–0.68, *p* < 0.001), 42% (OR = 0.58, 95% CI: 0.32–0.98, *p* = 0.050) and 63% (OR = 0.37, 95% CI: 0.23–0.59, *p* < 0.001), respectively when compared to Algerian participants. Also, the odds of moderate level of knowledge decreased significantly among participants with diploma or trade qualification, primary and secondary, intermediate, higher secondary education by 19% (OR = 0.81, 95% CI: 0.66–0.98, *p* = 0.034), 78% (OR = 0.22, 95% CI: 0.05–0.59, *p* = 0.010) and 35% (OR = 0.65, 95% CI: 0.53–0.79, *p* < 0.001), respectively when compared to participants with bachelors, masters or doctorate degree.

Similar to the odds of high level of knowledge, the odds of moderate level of knowledge increased significantly among participants working in the medical field by about 2.6 folds (OR = 2.60, 95% CI: 2.24–3.03, *p* < 0.001) and participants working as sports coach by 71% (OR = 1.71, 95% CI: 1.38–2.09, *p* < 0.001) when compared other participants. who doesn't On the contrary, the odds of moderate level of knowledge decreased significantly among unemployed participants by about 48% (OR = 0.52, 95% CI: 0.36–0.73, *p* < 0.001) when compared to participants working in government sector. While the odds of moderate level of knowledge increased significantly by about 1% for each one unite increase in the participants height (OR = 1.01, 95%CI: (1.00–1.01), *p* = 0.026).

Notably, the odds of moderate level of knowledge level increased significantly among participants who used only nutritional supplements by nearly 58% (OR = 1.58, 95% CI: 1.07–2.40, *p* = 0.025) when compared to participants who used both anabolic hormones and nutritional supplements (Table 6).

**Table 6:** Logistic regression models for the association between demographics and moderate level of knowledge.

**Demographics**		**Low**	**Moderate**	**OR (univariable)**	**OR (multivariable)**
Age	Mean (SD)	27.3 (8.7)	27.0 (8.1)	0.99 (0.99–1.00, *p* = 0.252)	1.01 (0.99–1.02, *p* = 0.331)
Sex	Female	1,789 (82.6)	377 (17.4)	-	-
	Male	2,562 (84.0)	489 (16.0)	0.91 (0.78–1.05, *p* = 0.188)	0.86 (0.70–1.06, *p* = 0.166)
Country of residence	Algeria	337 (80.0)	84 (20.0)	-	-
	Bahrain	136 (85.5)	23 (14.5)	0.68 (0.40–1.10, *p* = 0.130)	0.92 (0.53–1.55, *p* = 0.762)
	Egypt	357 (76.8)	108 (23.2)	1.21 (0.88–1.68, *p* = 0.238)	1.00 (0.70–1.42, *p* = 0.989)
	Iraq	331 (84.2)	62 (15.8)	0.75 (0.52–1.08, *p* = 0.121)	0.93 (0.62–1.38, *p* = 0.716)
	Jordan	236 (75.2)	78 (24.8)	1.33 (0.93–1.88, *p* = 0.114)	1.19 (0.81–1.74, *p* = 0.380)
	Qatar	43 (89.6)	5 (10.4)	0.47 (0.16–1.11, *p* = 0.118)	0.65 (0.21–1.62, *p* = 0.395)
	Kuwait	303 (83.2)	61 (16.8)	0.81 (0.56–1.16, *p* = 0.251)	1.03 (0.67–1.59, *p* = 0.890)
	Lebanon	307 (79.5)	79 (20.5)	1.03 (0.73–1.46, *p* = 0.856)	1.22 (0.83–1.78, *p* = 0.310)
	Libya	116 (82.3)	25 (17.7)	0.86 (0.52–1.40, *p* = 0.564)	0.84 (0.48–1.40, *p* = 0.507)
	Morocco	431 (85.3)	74 (14.7)	0.69 (0.49–0.97, *p* = 0.033)	0.89 (0.61–1.30, *p* = 0.557)
	Oman	1 (100.0)	0 (0.0)	0.00 (NA-Inf, *p* = 0.982)	-
	Palestine	337 (86.4)	53 (13.6)	0.63 (0.43–0.92, *p* = 0.016)	0.67 (0.45–1.00, *p* = 0.049)
	Saudi	347 (87.0)	52 (13.0)	0.60 (0.41–0.87, *p* = 0.008)	0.72 (0.46–1.11, *p* = 0.141)
	Sudan	377 (89.8)	43 (10.2)	0.46 (0.31–0.68, *p* < 0.001)	0.44 (0.29–0.66, *p* < 0.001)
	Syria	294 (79.5)	76 (20.5)	1.04 (0.73–1.47, *p* = 0.837)	1.00 (0.69–1.45, *p* = 0.996)
	Tunisia	3 (100.0)	0 (0.0)	0.00 (NA-Inf, *p* = 0.969)	0.00 (NA-Inf, *p* = 0.954)
	UAE	125 (87.4)	18 (12.6)	0.58 (0.32–0.98, *p* = 0.050)	0.75 (0.39–1.37, *p* = 0.363)
	Yemen	270 (91.5)	25 (8.5)	0.37 (0.23–0.59, *p* < 0.001)	0.40 (0.23–0.65, *p* < 0.001)
Geographic location	Rural	634 (85.1)	111 (14.9)	-	-
	Urban	3,717 (83.1)	755 (16.9)	1.16 (0.94–1.45, *p* = 0.178)	1.22 (0.97–1.55, *p* = 0.093)
Your highest educational level	Bachelors/Masters/Doctorate	2,564 (81.5)	581 (18.5)	-	-
	Diploma/Trade Qualification	793 (84.5)	145 (15.5)	0.81 (0.66–0.98, *p* = 0.034)	0.84 (0.68–1.04, *p* = 0.119)
	Primary	61 (95.3)	3 (4.7)	0.22 (0.05–0.59, *p* = 0.010)	0.36 (0.09–1.02, *p* = 0.095)
	Secondary/Intermediate/ Higher Secondary	933 (87.2)	137 (12.8)	0.65 (0.53–0.79, *p* < 0.001)	0.76 (0.60–0.95, *p* = 0.016)
Do you work in the medical field?	No	3,368 (87.3)	492 (12.7)	-	-
	Yes	983 (72.4)	374 (27.6)	2.60 (2.24–3.03, *p* < 0.001)	2.54 (2.14–3.01, *p* < 0.001)
Are you a sports coach?	No	3,916 (84.3)	728 (15.7)	-	-
	Yes	435 (75.9)	138 (24.1)	1.71 (1.38–2.09, *p* < 0.001)	1.91 (1.51–2.42, *p* < 0.001)
Where do you work?	Government Sector	808 (82.0)	177 (18.0)	-	-
	Housewife	174 (84.9)	31 (15.1)	0.81 (0.53–1.22, *p* = 0.329)	1.34 (0.83–2.12, *p* = 0.221)
	Private Sector	1,250 (83.9)	240 (16.1)	0.88 (0.71–1.09, *p* = 0.226)	0.92 (0.72–1.17, *p* = 0.484)
	Student	1,714 (82.2)	372 (17.8)	0.99 (0.81–1.21, *p* = 0.927)	1.17 (0.90–1.54, *p* = 0.246)
	Unemployed	405 (89.8)	46 (10.2)	0.52 (0.36–0.73, *p* < 0.001)	0.75 (0.51–1.09, *p* = 0.142)
Marital state	Divorced	71 (83.5)	14 (16.5)	-	-
	Married	1,196 (84.9)	213 (15.1)	0.90 (0.52–1.70, *p* = 0.736)	0.74 (0.41–1.43, *p* = 0.344)
	Single	3,054 (82.7)	637 (17.3)	1.06 (0.61–1.97, *p* = 0.849)	0.78 (0.43–1.52, *p* = 0.445)
	Widowed	30 (93.8)	2 (6.2)	0.34 (0.05–1.31, *p* = 0.168)	0.33 (0.05–1.33, *p* = 0.165)
Your monthly income in dollar	Mean (SD)	730.9 (1,473.2)	641.6 (1,261.3)	1.00 (1.00–1.00, *p* = 0.100)	1.00 (1.00–1.00, *p* = 0.318)
Weight before COVID-19 lockdown, Kg	Mean (SD)	72.7 (17.9)	73.9 (20.3)	1.00 (1.00–1.01, *p* = 0.088)	1.01 (1.00–1.01, *p* = 0.071)
Weight during COVID-19 lcokdown, Kg	Mean (SD)	73.8 (20.9)	74.3 (17.6)	1.00 (1.00–1.00, *p* = 0.525)	1.00 (0.99–1.00, *p* = 0.330)
Height, cm	Mean (SD)	168.0 (18.4)	169.5 (13.5)	1.01 (1.00–1.01, *p* = 0.026)	1.00 (1.00–1.01, *p* = 0.207)
Fat percentage before COVID-19 lockdown	Mean (SD)	19.0 (7.4)	18.9 (7.3)	1.00 (0.99–1.01, *p* = 0.648)	1.00 (0.98–1.02, *p* = 0.990)
Fat percentage during COVID-19 lockdown	Mean (SD)	19.9 (7.8)	19.7 (7.6)	1.00 (0.99–1.01, *p* = 0.436)	1.00 (0.98–1.02, *p* = 0.950)
Do you smoke?	No	3,361 (83.1)	682 (16.9)	-	-
	Yes	990 (84.3)	184 (15.7)	0.92 (0.77–1.09, *p* = 0.332)	0.98 (0.79–1.19, *p* = 0.810)
Do you use hormones and supplements?	Both of them	193 (85.8)	32 (14.2)	-	-
	Hormones only	32 (84.2)	6 (15.8)	1.13 (0.40–2.76, *p* = 0.799)	1.86 (0.63–4.82, *p* = 0.226)
	None of them	3,288 (84.4)	608 (15.6)	1.12 (0.77–1.67, *p* = 0.578)	1.36 (0.89–2.15, *p* = 0.170)
	Supplements only	838 (79.2)	220 (20.8)	1.58 (1.07–2.40, *p* = 0.025)	1.91 (1.23–3.05, *p* = 0.005)

### Adjusted logistic regression model for the association between demographics and moderate level of knowledge

The adjusted odds of moderate level of knowledge decreased significantly among participants from Palestine, Sudan and Yemen by about 33% (OR = 0.67, 95%CI: (0.45–1.00, *p* = 0.049), 56% (OR = 0.44, 95%CI: (0.29–0.66, *p* < 0.001) and 60% (OR = 0.40, 95%CI: (0.23–0.65, *p* < 0.001), respectively, compared to the Algerian participants. It decreased significantly among participants with secondary, intermediate, or higher secondary education by 24% (OR = 0.76, 95%CI: (0.60–0.95, *p* = 0.016) when compared to participants with bachelors, masters, or doctorate degree.

Again, the adjusted odds of moderate level of knowledge level increased significantly among participants working in the medical field by about 2.5 folds (OR = 2.54, 95%CI: (2.14–3.01, *p* < 0.001) and those working as sport coach by 91% (OR = 1.91, 95%CI: (1.51–2.42, *p* < 0.001) when compared to other participants. It also increased significantly among participants who used only nutritional supplements by 91% (OR = 1.91, 95%CI: (1.23–3.05, *p* = 0.005) in comparison to participants who used both anabolic hormones and nutritional supplements (Table 6).

### Univariate logistic regression model for the association between demographics and high level of attitude

The odds of high level of attitude increased significantly among males by about 60% (OR = 1.60, 95% CI: 1.19–2.18, *p* = 0.002) compared to females. It increased significantly among participants from Jordan and Sudan by 2.2 folds (OR = 2.15, 95% CI: 1.07–4.47, *p* = 0.034) and 3.9 folds (OR = 3.87, 95% CI: 2.11–7.61, *p* < 0.001), respectively, compared to the Algerian participants. While the odds of high level of attitude level decreased significantly among the urban participants by 40% (OR = 0.60, 95% CI: 0.43–0.85, *p* = 0.003) compared to the rural participants. It also decreased significantly among participants with secondary, intermediate or higher secondary education by 33% (OR = 0.67, 95% CI: 0.44–0.98, *p* = 0.048) compared to participants with bachelors, masters or doctorate degree.

While the odds of high level of attitude increased significantly among participants working in the medical field by about 71% (OR = 1.71, 95% CI: 1.28–2.28, *p* < 0.001) compared to others. Also, it increased significantly among participants who used only nutritional supplements by 4 folds (OR = 4.04, 95% CI: 2.00–9.67, *p* < 0.001) compared to participants who used both anabolic hormones and nutritional supplements (Table 7).

**Table 7:** Logistic regression models for the association between demographics and high level of attitude.

**Demographics**		**Low**	**High**	**OR (univariable)**	**OR (multivariable)**
Age	Mean (SD)	27.3 (8.6)	27.0 (7.4)	1.00 (0.98–1.01, *p* = 0.619)	0.98 (0.96–1.01, *p* = 0.286)
Sex	Female	2,085 (97.1)	63 (2.9)	-	-
	Male	2,933 (95.4)	142 (4.6)	1.60 (1.19–2.18, *p* = 0.002)	1.38 (0.91–2.11, *p* = 0.137)
Residence	Algeria	410 (96.9)	13 (3.1)	-	-
	Bahrain	151 (93.8)	10 (6.2)	2.09 (0.87–4.85, *p* = 0.088)	1.99 (0.78–4.97, *p* = 0.141)
	Egypt	459 (97.2)	13 (2.8)	0.89 (0.41–1.97, *p* = 0.777)	0.86 (0.36–2.03, *p* = 0.723)
	Iraq	384 (98.2)	7 (1.8)	0.57 (0.21–1.42, *p* = 0.243)	0.65 (0.21–1.77, *p* = 0.410)
	Jordan	308 (93.6)	21 (6.4)	2.15 (1.07–4.47, *p* = 0.034)	2.02 (0.95–4.47, *p* = 0.074)
	Qatar	52 (92.9)	4 (7.1)	2.43 (0.66–7.15, *p* = 0.133)	2.43 (0.61–8.06, *p* = 0.168)
	Kuwait	357 (96.0)	15 (4.0)	1.33 (0.62–2.86, *p* = 0.466)	2.52 (1.05–6.13, *p* = 0.038)
	Lebanon	384 (96.2)	15 (3.8)	1.23 (0.58–2.66, *p* = 0.588)	1.37 (0.60–3.19, *p* = 0.452)
	Libya	137 (95.8)	6 (4.2)	1.38 (0.48–3.57, *p* = 0.521)	1.20 (0.40–3.27, *p* = 0.729)
	Morocco	509 (98.1)	10 (1.9)	0.62 (0.26–1.42, *p* = 0.261)	0.88 (0.34–2.20, *p* = 0.793)
	Oman	1 (100.0)	0 (0.0)	0.00 (NA-Inf, *p* = 0.985)	-
	Palestine	378 (96.7)	13 (3.3)	1.08 (0.49–2.39, *p* = 0.838)	1.05 (0.45–2.45, *p* = 0.911)
	Saudi	363 (96.3)	14 (3.7)	1.22 (0.56–2.65, *p* = 0.617)	1.84 (0.78–4.39, *p* = 0.165)
	Sudan	350 (89.1)	43 (10.9)	3.87 (2.11–7.61, *p* < 0.001)	4.78 (2.46–9.94, *p* < 0.001)
	Syria	373 (97.9)	8 (2.1)	0.68 (0.27–1.62, *p* = 0.390)	0.70 (0.26–1.77, *p* = 0.456)
	Tunisia	1 (100.0)	0 (0.0)	0.00 (NA-Inf, *p* = 0.985)	0.00 (NA-Inf, *p* = 0.997)
	UAE	131 (94.9)	7 (5.1)	1.69 (0.62–4.20, *p* = 0.276)	1.60 (0.50–4.61, *p* = 0.401)
	Yemen	270 (97.8)	6 (2.2)	0.70 (0.24–1.80, *p* = 0.477)	0.99 (0.32–2.77, *p* = 0.990)
Geographic	Rural	702 (94.1)	44 (5.9)	-	-
	Urban	4,316 (96.4)	161 (3.6)	0.60 (0.43–0.85, *p* = 0.003)	0.46 (0.31–0.68, *p* < 0.001)
Your highest educational level	Bachelor/Master/PhD	3062 (95.7)	138 (4.3)	-	-
	Diploma/Trade Qualification	892 (96.0)	37 (4.0)	0.92 (0.63–1.32, *p* = 0.661)	1.12 (0.74–1.67, *p* = 0.581)
	Primary	66 (100.0)	0 (0.0)	0.00 (0.00–0.09, *p* = 0.964)	0.00 (0.00–0.00, *p* = 0.976)
	Secondary/Intermediate/ Higher Secondary	998 (97.1)	30 (2.9)	0.67 (0.44–0.98, *p* = 0.048)	1.01 (0.63–1.58, *p* = 0.956)
Do you work in the medical field?	No	3,672 (96.7)	126 (3.3)	-	-
	Yes	1,346 (94.5)	79 (5.5)	1.71 (1.28–2.28, *p* < 0.001)	1.98 (1.40–2.79, *p* < 0.001)
Are you a sports coach?	No	4,444 (96.1)	178 (3.9)	-	-
	Yes	574 (95.5)	27 (4.5)	1.17 (0.76–1.75, *p* = 0.447)	0.85 (0.52–1.35, *p* = 0.499)
Where do you work?	Government Sector	955 (95.7)	43 (4.3)	-	-
	Housewife	199 (96.6)	7 (3.4)	0.78 (0.32–1.65, *p* = 0.552)	1.22 (0.46–2.91, *p* = 0.671)
	Private Sector	1,415 (94.6)	80 (5.4)	1.26 (0.86–1.85, *p* = 0.240)	1.10 (0.71–1.73, *p* = 0.673)
	Student	2,029 (97.0)	63 (3.0)	0.69 (0.47–1.03, *p* = 0.065)	0.71 (0.42–1.20, *p* = 0.197)
	Unemployed	420 (97.2)	12 (2.8)	0.63 (0.32–1.18, *p* = 0.170)	0.72 (0.33–1.46, *p* = 0.380)
Marital state	Divorced	75 (97.4)	2 (2.6)	-	-
	Married	1,351 (95.4)	65 (4.6)	1.80 (0.55–11.12, *p* = 0.417)	1.45 (0.41–9.26, *p* = 0.621)
	Single	3,562 (96.3)	138 (3.7)	1.45 (0.45–8.89, *p* = 0.605)	0.87 (0.24–5.63, *p* = 0.851)
	Widowed	30 (100.0)	0 (0.0)	0.00 (NA-Inf, *p* = 0.976)	0.00 (NA-Inf, *p* = 0.983)
Your monthly income in dollar.	Mean (SD)	709.0 (1,434.5)	744.8 (1,360.5)	1.00 (1.00–1.00, *p* = 0.727)	1.00 (1.00–1.00, *p* = 0.224)
Weight.before.COVID.19.era.Kg.	Mean (SD)	73.1 (18.5)	74.9 (14.9)	1.00 (1.00–1.01, *p* = 0.157)	1.00 (0.99–1.01, *p* = 0.752)
Weight.during.COVID.19.era.Kg.	Mean (SD)	74.2 (20.7)	77.0 (37.5)	1.00 (1.00–1.01, *p* = 0.073)	1.00 (0.99–1.01, *p* = 0.367)
Height.cm.	Mean (SD)	168.3 (17.7)	170.6 (21.4)	1.01 (1.00–1.02, *p* = 0.064)	1.00 (0.99–1.01, *p* = 0.440)
Fat Percentage before COVID-19 lockdown	Mean (SD)	19.0 (7.4)	18.5 (6.9)	0.99 (0.97-1.01, *p* = 0.280)	1.00 (0.96-1.04, *p* = 0.992)
Fat Percentage during COVID-19 lockdown	Mean (SD)	19.9 (7.8)	19.3 (7.4)	0.99 (0.97–1.01, *p* = 0.256)	0.99 (0.96–1.02, *p* = 0.594)
Do you smoke?	No	3,890 (96.2)	153 (3.8)	-	-
	Yes	1,128 (95.6)	52 (4.4)	1.17 (0.84–1.61, *p* = 0.333)	0.95 (0.65–1.37, *p* = 0.784)
Do you use hormones and supplements?	Both of them	217 (96.9)	7 (3.1)	-	-
	Hormones only	43 (97.7)	1 (2.3)	0.72 (0.04–4.20, *p* = 0.762)	1.11 (0.06–7.13, *p* = 0.925)
	None of them	3,845 (98.0)	78 (2.0)	0.63 (0.31–1.51, *p* = 0.247)	0.77 (0.34–2.08, *p* = 0.566)
	Supplements only	913 (88.5)	119 (11.5)	4.04 (2.00–9.67, *p* < 0.001)	5.21 (2.37–13.80, *p* < 0.001)

### Adjusted logistic regression model for the association between demographics and high level of attitude

The adjusted odds of high level of attitude increased significantly among participants from Kuwait and Sudan by 2.5 folds (OR = 2.52, 95%CI: (1.05–6.13, *p* = 0.038) and 4.8 folds (OR = 4.78, 95%CI: (2.46–9.94, *p* < 0.001), respectively, compared to Algerian participants; while it decreased significantly among the urban participants by 54% (OR = 0.46, 95%CI: (0.31–0.68, *p* < 0.001) in comparison to the rural participants.

Also, the adjusted odds of high level of attitude increased significantly among participants working in the medical field by about 98% (OR = 1.98, 95%CI: (1.40–2.79, *p* < 0.001) compared to other participants. It also increased significantly among participants who used only nutritional supplements by 5.2 folds (OR = 5.21, 95%CI: (2.37–13.80, *p* < 0.001) compared to participants who used both, anabolic hormones and nutritional supplements (Table 7).

### Univariate logistic regression model for the association between demographics and moderate level of attitude

The odds of moderate level of attitude increased significantly by 1% for each one-year increase in participant's age (OR = 1.01, 95% CI: 1.00–1.02, *p* = 0.027) while decreased significantly among males by 21% in comparison to females (OR = 0.79, 95% CI: 0.67–0.94, *p* = 0.007). Also, the odds of moderate level of attitude increased significantly among participants from Egypt, Jordan, Qatar, Kuwait, Lebanon, Palestine, Saudi, Sudan, Tunisia and UAE by about 99% (OR = 1.99, 95% CI: 1.28–3.17, *p* = 0.003), 2 folds (OR = 2.04, 95% CI: 1.27–3.34, *p* = 0.004), 2.6 folds (OR = 2.63, 95% CI: 1.16-5.53, *p* = 0.014), 2.3 folds (OR = 2.26, 95% CI: 1.43–3.62, *p* = 0.001), 78% (OR = 1.78, 95% CI: 1.12–2.88, *p* = 0.017), 70% (OR = 1.70, 95% CI: 1.06–2.77, *p* = 0.030), 2.3 folds (OR = 2.30, 95% CI: 1.46–3.68, *p* < 0.001), 2 folds (OR = 2.03, 95% CI: 1.28–3.29, *p* = 0.003), 27.3 folds (OR = 27.33, 95% CI: 2.55–598.16, *p* = 0.008) and 3.2 folds (OR = 3.23, 95% CI: 1.88–5.56, *p* < 0.001), respectively, compared to the Algerian participants.

Also, the odds of moderate level of attitude increased significantly among participants working in the medical field by about 20% (OR = 1.20, 95% CI: 1.00–1.44), *p* = 0.049) compared to others; but it decreased significantly among participants working in private sector by 26% (OR = 0.74, 95% CI: 0.58–0.94, *p* = 0.014) compared to participants working in government Sector and among married and single participants by 41% (OR = 0.59, 95% CI: 0.35–1.03, *p* = 0.051) and 52% (OR = 0.48, 95% CI: 0.29–0.83, *p* = 0.006), respectively, compared to divorced participants.

The odds of moderate level of attitude increased significantly among participants who did not use either anabolic hormones or nutritional supplements and participants who used only supplements by 70% (OR = 1.70, 95% CI: 1.05–2.96, *p* = 0.045) and 84% (OR = 1.84, 95% CI: 1.10–3.28, *p* = 0.027), respectively compared to participants who used both (Table 8).

**Table 8:** Logistic regression models for the association between demographics and moderate level of attitude.

**Demographics**		**Low**	**Moderate**	**OR (univariable)**	**OR (multivariable)**
Age	Mean (SD)	27.3 (8.6)	28.1 (8.8)	1.01 (1.00–1.02, *p* = 0.027)	1.01 (0.99–1.02, *p* = 0.466)
Sex	Female	2,085 (87.6)	294 (12.4)	-	-
	Male	2,933 (89.9)	328 (10.1)	0.79 (0.67–0.94, *p* = 0.007)	0.93 (0.74–1.16, *p* = 0.495)
Country of residence	Algeria	410 (93.2)	30 (6.8)	-	-
	Bahrain	151 (89.3)	18 (10.7)	1.63 (0.87–2.98, *p* = 0.119)	1.52 (0.79–2.83, *p* = 0.197)
	Egypt	459 (87.3)	67 (12.7)	1.99 (1.28–3.17, *p* = 0.003)	1.74 (1.10–2.82, *p* = 0.021)
	Iraq	384 (92.1)	33 (7.9)	1.17 (0.70–1.97, *p* = 0.539)	1.12 (0.66–1.93, *p* = 0.675)
	Jordan	308 (87.0)	46 (13.0)	2.04 (1.27–3.34, *p* = 0.004)	1.91 (1.15–3.19, *p* = 0.013)
	Qatar	52 (83.9)	10 (16.1)	2.63 (1.16–5.53, *p* = 0.014)	2.33 (1.00–5.08, *p* = 0.040)
	Kuwait	357 (85.8)	59 (14.2)	2.26 (1.43–3.62, *p* = 0.001)	1.95 (1.18–3.29, *p* = 0.010)
	Lebanon	384 (88.5)	50 (11.5)	1.78 (1.12–2.88, *p* = 0.017)	1.77 (1.09–2.94, *p* = 0.024)
	Libya	137 (93.8)	9 (6.2)	0.90 (0.39–1.87, *p* = 0.784)	0.92 (0.40–1.94, *p* = 0.835)
	Morocco	509 (91.9)	45 (8.1)	1.21 (0.75–1.97, *p* = 0.440)	1.17 (0.72–1.94, *p* = 0.534)
	Oman	1 (100.0)	0 (0.0)	0.00 (NA-Inf, *p* = 0.964)	-
	Palestine	378 (88.9)	47 (11.1)	1.70 (1.06–2.77, *p* = 0.030)	1.51 (0.93–2.50, *p* = 0.099)
	Saudi	363 (85.6)	61 (14.4)	2.30 (1.46–3.68, *p* < 0.001)	1.84 (1.12–3.08, *p* = 0.018)
	Sudan	350 (87.1)	52 (12.9)	2.03 (1.28–3.29, *p* = 0.003)	2.05 (1.27–3.36, *p* = 0.004)
	Syria	373 (92.3)	31 (7.7)	1.14 (0.67–1.92, *p* = 0.632)	1.11 (0.65–1.90, *p* = 0.709)
	Tunisia	1 (33.3)	2 (66.7)	27.33 (2.55–598.16, *p* = 0.008)	25.65 (2.36–565.82, *p* = 0.009)
	UAE	131 (80.9)	31 (19.1)	3.23 (1.88–5.56, *p* < 0.001)	2.68 (1.48–4.85, *p* = 0.001)
	Yemen	270 (89.7)	31 (10.3)	1.57 (0.93–2.66, *p* = 0.093)	1.40 (0.80–2.45, *p* = 0.233)
Geographic location	Rural	702 (88.6)	90 (11.4)	-	-
	Urban	4,316 (89.0)	532 (11.0)	0.96 (0.76–1.23, *p* = 0.745)	0.90 (0.70–1.16, *p* = 0.417)
Your highest educational level	Bachelors/Masters/Doctorate	3,062 (88.5)	397 (11.5)	-	-
	Diploma/Trade Qualification	892 (90.2)	97 (9.8)	0.84 (0.66–1.06, *p* = 0.141)	0.95 (0.74–1.21, *p* = 0.689)
	Primary	66 (100.0)	0 (0.0)	0.00 (0.00–0.03, *p* = 0.961)	0.00 (0.00–0.00, *p* = 0.943)
	Secondary/Intermediate/ Higher Secondary	998 (88.6)	128 (11.4)	0.99 (0.80–1.22, *p* = 0.920)	1.10 (0.87–1.39, *p* = 0.415)
Do you work in the medical field?	No	3,672 (89.5)	432 (10.5)	-	-
	Yes	1346 (87.6)	190 (12.4)	1.20 (1.00–1.44, *p* = 0.049)	1.25 (1.02–1.53, *p* = 0.028)
Are you a sports coach?	No	4,444 (88.8)	560 (11.2)	-	-
	Yes	574 (90.3)	62 (9.7)	0.86 (0.64–1.12, *p* = 0.274)	1.05 (0.77–1.40, *p* = 0.768)
Where do you work?	Government Sector	955 (87.2)	140 (12.8)	-	-
	Housewife	199 (87.7)	28 (12.3)	0.96 (0.61–1.46, *p* = 0.853)	0.85 (0.52–1.37, *p* = 0.522)
	Private Sector	1,415 (90.2)	153 (9.8)	0.74 (0.58–0.94, *p* = 0.014)	0.81 (0.62–1.06, *p* = 0.128)
	Student	2,029 (89.3)	242 (10.7)	0.81 (0.65–1.02, *p* = 0.068)	0.97 (0.72–1.31, *p* = 0.847)
	Unemployed	420 (87.7)	59 (12.3)	0.96 (0.69–1.32, *p* = 0.797)	1.15 (0.79–1.65, *p* = 0.460)
Marital state	Divorced	75 (80.6)	18 (19.4)	-	-
	Married	1,351 (87.7)	190 (12.3)	0.59 (0.35–1.03, *p* = 0.051)	0.57 (0.33–1.03, *p* = 0.049)
	Single	3,562 (89.7)	408 (10.3)	0.48 (0.29–0.83, *p* = 0.006)	0.47 (0.27–0.87, *p* = 0.012)
	Widowed	30 (83.3)	6 (16.7)	0.83 (0.28–2.21, *p* = 0.725)	0.76 (0.24–2.15, *p* = 0.621)
Your monthly income in dollar	Mean (SD)	709.0 (1,434.5)	987.1 (1,777.1)	1.00 (1.00–1.00, *p* < 0.001)	1.00 (1.00–1.00, *p* = 0.169)
Weight before COVID-19 lockdown, Kg	Mean (SD)	73.1 (18.5)	72.2 (16.1)	1.00 (0.99–1.00, *p* = 0.245)	1.00 (0.99–1.01, *p* = 0.500)
Weight during COVID-19 lockdown, Kg	Mean (SD)	74.2 (20.7)	73.0 (16.0)	1.00 (0.99–1.00, *p* = 0.181)	1.00 (0.99–1.01, *p* = 0.784)
Height, cm	Mean (SD)	168.3 (17.7)	167.6 (16.0)	1.00 (0.99–1.00, *p* = 0.343)	1.00 (1.00–1.01, *p* = 0.822)
Fat percentage before COVID-19 lcokdown	Mean (SD)	19.0 (7.4)	19.4 (7.5)	1.01 (1.00–1.02, *p* = 0.207)	1.01 (0.98–1.03, *p* = 0.654)
Fat percentage during COVID-19 lockdown	Mean (SD)	19.9 (7.8)	20.3 (8.0)	1.01 (1.00–1.02, *p* = 0.283)	1.00 (0.98–1.02, *p* = 0.850)
Do you smoke?	No	3,890 (88.8)	490 (11.2)	-	-
	Yes	1,128 (89.5)	132 (10.5)	0.93 (0.76–1.14, *p* = 0.478)	1.00 (0.79–1.25, *p* = 0.974)
Do you use hormones and supplements?	Both of them	217 (93.1)	16 (6.9)	-	-
	Hormones only	43 (97.7)	1 (2.3)	0.32 (0.02–1.61, *p* = 0.269)	0.30 (0.02–1.60, *p* = 0.259)
	None of them	3,845 (88.9)	481 (11.1)	1.70 (1.05–2.96, *p* = 0.045)	1.53 (0.90–2.78, *p* = 0.139)
	Supplements only	913 (88.0)	124 (12.0)	1.84 (1.10–3.28, *p* = 0.027)	1.72 (1.00–3.18, *p* = 0.065)

### Adjusted logistic regression model for the association between demographics and moderate level of attitude

The adjusted odds of moderate level of attitude increased significantly among participants from Egypt, Jordan, Qatar, Kuwait, Lebanon, Saudi, Sudan, Tunisia and UAE by 74% (OR = 1.74, 95%CI: (1.10–2.82, *p* = 0.021), 91% (OR = 1.91, 95%CI: (1.15–3.19, *p* = 0.013), 2.3 folds (OR = 2.33, 95%CI: (1.00–5.08, *p* = 0.040), 95% (OR = 1.95, 95%CI: (1.18–3.29, *p* = 0.010), 77% (OR = 1.77, 95%CI: (1.09–2.94, *p* = 0.024), 84% (OR = 1.84, 95%CI: (1.12–3.08, *p* = 0.018), 2 folds (OR = 2.05, 95%CI: (1.27–3.36, *p* = 0.004), 25.7 folds (OR = 25.65, 95%CI: (2.36–565.82, *p* = 0.009), and 2.7 folds (OR = 2.68, 95%CI: (1.48–4.85, *p* = 0.001), respectively compared to the Algerian participants.

Moreover, the adjusted odds of moderate level of attitude level increased significantly among participants working in the medical field by 25% compared to others (OR = 1.25, 95%CI: (1.02–1.53, *p* = 0.028). It decreased significantly among married and single participants by 43% (OR = 0.57, 95%CI: (0.33–1.03, *p* = 0.049) and 53% (OR = 0.47, 95%CI: (0.27–0.87, *p* = 0.012), respectively compared to divorced participants (Table 8).

### Univariate logistic regression model for the association between demographics and high level of practice

The odds of high level of practice increased significantly among males by about 3.4 folds compared to females (OR = 3.41, 95% CI: 1.60–8.40, *p* = 0.003). It increased significantly among sport coaches by about 8 folds compared to participants who are not sport coaches (OR = 8.03, 95% CI: 4.28–15.07, *p* < 0.001). The odds of high level of practice level increased significantly by about 1% for each one kg increase in participants' weight before (OR = 1.01, 95% CI: 1.00–1.02, *p* = 0.001) and during COVID-19 lockdown (OR = 1.01, 95% CI: 1.00–1.01, *p* = 0.041). Notably, the odds decreased significantly by 10% (OR = 0.90, 95% CI: 0.86–0.95, *p* < 0.001) and 9% (OR = 0.91, 95% CI: 0.87–0.95, *p* < 0.001) for each one unit increase in participants' fat percentage before and during COVD-19 lockdown, respectively. The odds also decreased significantly among participants who used only nutritional supplements by 82% compared to participants who used both anabolic hormones and nutritional supplements (OR = 0.18, 95% CI: 0.10–0.35, *p* < 0.001) (Table 9).

**Table 9:** Logistic regression models for the association between demographics and high level of practice.

**Demographics**		**Low**	**High**	**OR (univariable)**	**OR (multivariable)**
Age	Mean (SD)	27.4 (8.6)	28.1 (6.2)	1.01 (0.97–1.04, *p* = 0.612)	1.04 (0.97–1.12, *p* = 0.241)
Sex	Female	2,435 (99.7)	7 (0.3)	-	-
	Male	3,370 (99.0)	33 (1.0)	3.41 (1.60–8.40, *p* = 0.003)	0.75 (0.23–2.65, *p* = 0.631)
Country of residence	Algeria	447 (98.7)	6 (1.3)	-	-
	Bahrain	179 (100.0)	0 (0.0)	0.00 (NA-Inf, *p* = 0.990)	0.00 (0.00-Inf, *p* = 0.993)
	Egypt	538 (99.8)	1 (0.2)	0.14 (0.01–0.81, *p* = 0.068)	0.45 (0.02–3.94, *p* = 0.507)
	Iraq	422 (99.5)	2 (0.5)	0.35 (0.05–1.54, *p* = 0.204)	0.70 (0.08–5.24, *p* = 0.728)
	Jordan	365 (97.3)	10 (2.7)	2.04 (0.75–6.05, *p* = 0.171)	3.20 (0.81–16.08, *p* = 0.116)
	Qatar	64 (97.0)	2 (3.0)	2.33 (0.34–10.35, *p* = 0.307)	5.91 (0.57–51.32, *p* = 0.109)
	Kuwait	428 (99.3)	3 (0.7)	0.52 (0.11–1.99, *p* = 0.360)	0.93 (0.10–7.45, *p* = 0.947)
	Lebanon	448 (99.8)	1 (0.2)	0.17 (0.01–0.98, *p* = 0.097)	1.15 (0.05–10.20, *p* = 0.907)
	Libya	147 (96.7)	5 (3.3)	2.53 (0.72–8.53, *p* = 0.129)	2.54 (0.51–14.40, *p* = 0.261)
	Morocco	563 (99.8)	1 (0.2)	0.13 (0.01–0.78, *p* = 0.062)	0.56 (0.03–5.06, *p* = 0.634)
	Oman	1 (100.0)	0 (0.0)	0.00 (NA-Inf, *p* = 0.999)	-
	Palestine	438 (100.0)	0 (0.0)	0.00 (NA-Inf, *p* = 0.985)	0.00 (0.00-Inf, *p* = 0.990)
	Saudi	437 (99.8)	1 (0.2)	0.17 (0.01–1.00, *p* = 0.102)	0.37 (0.01–4.02, *p* = 0.455)
	Sudan	440 (98.9)	5 (1.1)	0.85 (0.24–2.83, *p* = 0.785)	3.41 (0.73–18.77, *p* = 0.126)
	Syria	411 (99.8)	1 (0.2)	0.18 (0.01–1.07, *p* = 0.115)	0.72 (0.03–6.50, *p* = 0.786)
	Tunisia	3 (100.0)	0 (0.0)	0.00 (NA-Inf, *p* = 0.999)	0.00 (0.00-Inf, *p* = 0.999)
	UAE	169 (100.0)	0 (0.0)	0.00 (NA-Inf, *p* = 0.990)	0.00 (0.00-Inf, *p* = 0.994)
	Yemen	305 (99.3)	2 (0.7)	0.49 (0.07–2.14, *p* = 0.382)	2.71 (0.30–20.20, *p* = 0.331)
Geographic location	Rural	834 (99.8)	2 (0.2)	-	-
	Urban	4,971 (99.2)	38 (0.8)	3.19 (0.98–19.62, *p* = 0.110)	1.00 (0.25–6.76, *p* = 0.997)
Your highest educational level	Bachelor/Master/PhD	3,571 (99.3)	26 (0.7)	-	-
	Diploma/Trade Qualification	1,015 (98.9)	11 (1.1)	1.49 (0.70–2.95, *p* = 0.271)	1.46 (0.58–3.47, *p* = 0.400)
	Primary	66 (100.0)	0 (0.0)	0.00 (NA-Inf, *p* = 0.986)	0.00 (0.00-Inf, *p* = 0.996)
	Secondary/Intermediate/ Higher Secondary	1,153 (99.7)	3 (0.3)	0.36 (0.09–1.02, *p* = 0.092)	0.53 (0.11–1.74, *p* = 0.341)
Do you work in the medical field?	No	4,199 (99.3)	31 (0.7)	-	-
	Yes	1,606 (99.4)	9 (0.6)	0.76 (0.34–1.53, *p* = 0.468)	0.90 (0.34–2.17, *p* = 0.820)
Are you a sports coach?	No	5,162 (99.6)	20 (0.4)	-	-
	Yes	643 (97.0)	20 (3.0)	8.03 (4.28–15.07, *p* < 0.001)	3.00 (1.34–6.74, *p* = 0.007)
Where do you work?	Government Sector	1,131 (99.4)	7 (0.6)	-	-
	Housewife	233 (99.6)	1 (0.4)	0.69 (0.04–3.92, *p* = 0.733)	2.56 (0.11–25.83, *p* = 0.468)
	Private Sector	1,627 (98.7)	21 (1.3)	2.09 (0.93–5.31, *p* = 0.093)	0.92 (0.32–2.82, *p* = 0.871)
	Student	2,324 (99.6)	10 (0.4)	0.70 (0.27–1.92, *p* = 0.462)	1.25 (0.33–4.99, *p* = 0.742)
	Unemployed	490 (99.8)	1 (0.2)	0.33 (0.02–1.86, *p* = 0.300)	0.47 (0.02–3.68, *p* = 0.534)
Marital state	Divorced	94 (98.9)	1 (1.1)	-	-
	Married	1,596 (99.4)	10 (0.6)	0.59 (0.11–10.87, *p* = 0.616)	1.37 (0.16–32.56, *p* = 0.800)
	Single	4,079 (99.3)	29 (0.7)	0.67 (0.14–11.97, *p* = 0.693)	2.54 (0.28–63.06, *p* = 0.470)
	Widowed	36 (100.0)	0 (0.0)	0.00 (NA-Inf, *p* = 0.984)	0.00 (0.00-Inf, *p* = 0.997)
Your monthly income in dollar	Mean (SD)	738.0 (1,473.5)	1,056.4 (1,636.1)	1.00 (1.00–1.00, *p* = 0.202)	1.00 (1.00–1.00, *p* = 0.436)
Weight before COVID-19 lockdown, Kg	Mean (SD)	73.0 (18.1)	84.3 (21.3)	1.01 (1.00–1.02, *p* = 0.001)	1.01 (0.99–1.02, *p* = 0.108)
Weight during COVID-19 lockdown, Kg	Mean (SD)	74.1 (21.2)	81.8 (14.3)	1.01 (1.00–1.01, *p* = 0.041)	1.00 (0.98–1.01, *p* = 0.901)
Height, cm	Mean (SD)	168.3 (17.6)	172.5 (17.7)	1.03 (1.00–1.06, *p* = 0.090)	1.00 (0.99–1.03, *p* = 0.814)
Fat percentage before COVID-19 lockdown	Mean (SD)	19.1 (7.4)	14.3 (7.3)	0.90 (0.86–0.95, *p* < 0.001)	0.99 (0.90–1.08, *p* = 0.798)
Fat percentage during COVID-19 lockdown	Mean (SD)	20.0 (7.8)	15.2 (8.3)	0.91 (0.87–0.95, *p* < 0.001)	0.97 (0.89–1.05, *p* = 0.445)
Do you smoke?	No	4,500 (99.3)	33 (0.7)	-	-
	Yes	1,305 (99.5)	7 (0.5)	0.73 (0.30–1.56, *p* = 0.454)	0.35 (0.12–0.89, *p* = 0.036)
Do you use hormones and supplements?	Both of them	220 (91.7)	20 (8.3)	-	-
	Hormones only	45 (100.0)	0 (0.0)	0.00 (NA-Inf, *p* = 0.987)	0.00 (0.00-Inf, *p* = 0.996)
	None of them	4,403 (100.0)	1 (0.0)	0.00 (0.00–0.01, *p* < 0.001)	0.00 (0.00–0.03, *p* < 0.001)
	Supplements only	1,137 (98.4)	19 (1.6)	0.18 (0.10–0.35, *p* < 0.001)	0.21 (0.09–0.47, *p* < 0.001)

### Adjusted logistic regression model for the association between demographics and the high level of practice

The adjusted odds of high level of practice increased significantly among sport coaches by about 3 folds compared to other participants (OR = 3.00, 95%CI: (1.34–6.74, *p* = 0.007); while the adjusted odds of high practice level decreased significantly among smokers by about 65% compared to non-smokers (OR = 0.35, 95%CI: (0.12–0.89, *p* = 0.036). It also decreased significantly among participants who used only supplements and participants who neither used hormones nor supplements by 79% (OR = 0.21, 95%CI: (0.09–0.47, *p* < 0.001) and 100% (OR = 0.00, 95%CI: (0.00–0.03, *p* < 0.001), respectively compared to participants who used both anabolic hormones and nutritional supplements (Table 9).

The proportion of participants who were advised to use anabolic hormones and nutritional supplements by doctor, nutritionist, pharmacist, trainer, internet, self and miscellaneous was 2.1, 2.3, 0.75, 5.1, 4.2, 0.68, and 4.7%, respectively. The source of information for the used anabolic hormones and nutritional supplements was trainer (11.7%), doctor (21.4%), friends (39.2%), and internet (64%). The reason for using these hormones and supplements included body building (8.2%), performance improvement (6.1%), protection from disease (2.9%), and weight loss (2.4%). About 31.6% of the participants thought that anabolic hormones and nutritional supplements help to win championships, 66.3% thought that these help them to look better, and almost all the participants thought that these help to make them athletic and strong.

Analysis of this survey revealed that 14.3% of participants used proteins, 1.3% used energy bars, 6.9% used vitamins, and 1.1% used sport drinks before COVID-19 lockdown. During the COVID-19 lockdown, 6.9% used proteins, 1.2% used energy bar (carbohydrate), 7.2% used vitamins and 0.9% used sport drinks. Interestingly, before COVID-19 lockdown, 6.5% of participants used anabolic steroids, 0.5% used insulin, 0.75% used growth hormone (GH) and 0.4% used cortisol and during the lockdown, 4.1% of the study population used anabolic steroids, 0.4% used insulin, 0.5% used GH and 0.1% used cortisol. Tablets and injects were used by 1.6 and 1.2% of participants for administration of hormones, 1.4% used both and 8.1% did not use either of the two. Before COVID-19 lockdown, the anabolic hormones and nutritional supplements were sourced from gym trainer (3.2%), online stores (3.4%), and pharmacy (5.7%); during the lockdown, these products were sourced by gym trainer (2.9%), online stores (3.9%), and pharmacy (5.5%). Stopping the use of anabolic hormones led to “fluctuations in mood and depression” (1.6%), anorexia (0.8%), anxiety and insomnia (1.1%), “decrease in fitness” (0.9%), muscle weakness (1.8%), “desire to return to hormones again” (1%). No symptoms were reported in 1.5% of participants, 7.6% reported not using the anabolic hormones and 0.7% reported that they never stopped using hormones (Supplementary Table S1).

McNemar's test was used for comparative analysis between types of supplements used before and during COVID-19 lockdown. It showed a statistically significant decrease in consumption of proteins, carbohydrates and sport drinks during COVID-19 lockdown and a statistically significant increase in consumption of vitamins during COVID-19 lockdown compared to that prior to the COVID-19 lockdown (*p* < 0.001) (Supplementary Table S2).

Supplementary Table S3 depicts a statistically significant decrease in the use of all types of hormones during COVID-19 lockdown compared to that prior to the COVID-19 lockdown (*p* < 0.001). Further, procurement of these hormones and supplements showed a statistically significant decrease from gym trainer or pharmacy and statistically significant increase from online sources during COVID-19 lockdown compared to the before lockdown time (*p* < 0.001) (Supplementary Table S4).

## Discussion

This study reports knowledge, practice, and attitude toward anabolic hormones and nutritional supplements among people who exercise in Arab countries. This is the first report comparing people's practice before and during the COVID-19 lockdown. In this study, the mean age of participants was 27.4 (SD = 8.6). Studies from different countries reported that their participants were also of the similar age group which showing that exercising and using hormones and supplements are more common in the young age (13, 14, 24, 25). Among 5845 participants, we found that 19.8% of participants were using nutritional supplements alone, 0.8% of participants were using anabolic hormones alone, and 4.1% were using both the products at the same time. Different studies from various Arab countries showed a high prevalence of anabolic hormones users. In the UAE and Kuwait, the prevalence of anabolic hormones users was about 22% (14, 26); in Jordan it was 26% (27); in Iran, it was13% (28); and 9.8% in Saudi Arabia (23). The possible causes for these variations could be the difference in sample sizes and under reporting of self-reported drug abuse where participants feel embarrassed to admit their use. Regarding the use of nutritional supplements, studies reported that the prevalence was 36 % in Lebanon (29), and 66.7% in Iran (30). In this study, the main aims of using these hormones ad supplements were bodybuilding and improving performance which is similar to the one conducted in 2021 in Iraq (24). Most of the study population used proteins (14.3%) and vitamins (6.9%) as sources of nutritional supplements and anabolic steroids (6.5%) as sources of hormones. This was comparable with the data reported by studies conducted in Saudi Arabia (2020) where the most commonly used nutritional supplement was proteins and the most commonly used hormone was steroids. However, another study conducted in 2018 in Kuwait reported growth hormone (79.4%) to be the most commonly used anabolic hormone which indicates that substance abuse is not limited to steroids (13, 23, 25).

Regarding knowledge, more than 70% of the participants had low knowledge about the harmful effects of unsupervised use of anabolic hormones and nutritional supplements. This was reported in other studies as well (14, 23). Participants from Egypt, Jordan, Qatar, Kuwait, Lebanon, and UAE showed significantly high level of knowledge compared to the participants from Algeria. Also, participants who work in the medical field or as sports coaches showed significantly increased level of knowledge level compared with other participants. This finding is logical and can be explained that the nature of those jobs can help participants gain more knowledge about the effect of using these hormones and supplements. More than half of the participants (64%) got their information from the internet, followed by their friends (39%) and doctors (21.4%) being their source of information. A study conducted in Saudi Arabia (2020) reported similar results but with a much less percentage; 13.6% considered the online source as the main source of information while 3.8% considered physicians as the main source (25). This finding indicates the importance of a carefully organized online campaign to increase awareness about the abuse of hormones and supplements.

Next, we observed high level of practice increased significantly among males compared to females. This may be attributed to cultural reasons as anabolic hormones are known to the public for their use among males to build their muscles. About 100% of the participants believed that hormones and supplements can make them athletic and strong and nearly 66% of the participants believed that these hormones and supplements help them to look better. Similar beliefs were reported by another study that hormones and supplements can increase muscle size and strength (14). In our report, we found pharmacy to be the main source for hormones and supplements which is different from the other reports where gym trainers were the main providers (26, 27). This could be because of the regulations forbidding the purchasing or selling anabolic hormones from sources other than pharmacies. Trainers were the most common people to advise the participants to use hormones and supplements. The same results were reported in study conducted in 2008 in the UAE (14). This is because trainers at gyms, without paying attention to the adverse effects of these substances, want their trainees to improve rapidly to gain more reputations.

Regarding the difference in habits of using vitamins between before and during COVID-19 lockdown, it was found that the usage significantly increased during the COVID-19 lock down compared to before the lockdown. The same finding was reported by a study conducted in Saudi Arabia (31). The main reason behind that might be the media that frequently advised the public to take vitamins to protect against the corona virus and to help in the treatment in case of being infected. The source from which the participants bought hormones and supplements during the COVID-19 lock down has changed significantly toward the online source. During the COVID-19, there has been a complete lockdown which led to online shopping being one of the alternative sources for buying these substances in most cases. As a result, the danger of unsupervised practice or non-prescribed substances is expected to be increased during this period.

Our findings encourage the need for educational programs through social media and mass media to address the potential effect of these substances on health. Participants who use these drugs achieve their goals by gaining weight and improving their body image which makes it difficult to change their behaviors. That is why, it is necessary to offer training courses and use a comprehensive approach to modify the public belief (13). Another possible solution can be directed toward health care providers by providing courses for them to have more knowledge to advise their patients. Also, sports coaches need to be educated about the effects of hormones and supplements as they represent a powerful reason for many participants to start using these drugs (24). This current study has some limitations. First, hormones and supplements were included together in the questions related to knowledge, practice, and attitude to overcome the participants' fear to answer questions related to anabolic hormones. Second, the survey was self-reported which might lead to some degree of reporting bias. Third, this survey did not investigate the dose of these substances. At last, the study cannot report the cause-effect relationship.

## Conclusion

This cross-sectional study reported the knowledge, practice, and attitude toward the use of anabolic hormones and nutritional supplements in the MENA region. The level of knowledge was low among most of the participants. High level of knowledge was reported among participants in the medical field and participants who were sports coaches. The level of practice was high among male participants and sports coaches. Proteins and steroids were the most used supplements and hormones respectively. The source of information was mainly internet and the main source of procuring the substances was the pharmacy. During the COVID-19 lockdown, there has been an increase in the use of vitamins. Campaigns through social media should be done to aware the population about the harmful effect of these substances. Also, courses should be available for health care providers and sports coaches.

## Data availability statement

The original contributions presented in the study are included in the article/Supplementary materials, further inquiries can be directed to the corresponding author.

## Ethics statement

The studies involving human participants were reviewed and approved by Institutional Review Board Committee (IRB) at the Sahel General Hospital, Lebanon. The patients/participants provided their written informed consent to participate in this study.

## The EARG Group (Eltewacy Arab Research Group)

Mohammed Moutaz Alshaghel^1^, Mohamad Omar Honeine^2^, Ahmad Mohamad Hejazi^3^, Samira Boulbaroud^4^, Said Ihbour^5^, Hinde EL Mouhi^6^, Amira Taha^7^, Hadeer Elfadly^8^, Dalia Zaafar^9^, Amira Mahmoud^10^, Abdallah Saieed^11^, Chiboub Fellah Saliha^12^, Adel Mouffokes^13^, Imed-eddine Badis^12^, Selma Nihel Klouche-Djedid^14^, Riham Ismail^15^, Bahaa Osman^2^, Hiba Omar El Badawi^16^, Mahmoud S. M. Shatat^17^, Malak Ayman Qawasmi^18^, Sewar A. Elejla^19^, Muhammad Hamadneh^20^, Mathaba Siddig^21^, Reem Mahjoub^21^, Mahmoud Saleh^22^, Fatima Shwgee Monaha Mohammed Alameen^23^, Hawa Ali Adroub Ali^22^, Mohammad Alnifise^24^, Mohammed Al-Balawi^25^, Rasha Gaber^26^, Ebraheem Albazee^24^, Abdallateef Rashed Alkandari^24^, Fatma Ridha^27^, Sabri Hammoud^28^, Sirwan Khalid Ahmed^29^, Luma S. Abdalbaqi^30^, Awat Alla Khdir^29^, Dalya Sadulah^31^, Ahmed AL-Ghrairi^31^, Moustafa Alhashemi^1^, Omar Alfatohi Aljondi^3^, Hassan Alhaj Ali^1^, Lina Almahmoud^24^, Nour Alhisah^32^, Haneen Al-Abbadi^33^, Radi Tofaha Alhusseini^34^, Aref Ali^35^, Abeer Hasan Sharafuddin^36^, Maha Abdo^37^, Hatem Sultan^38^, Ali Abdul Ghaffar^3^, Husain A.Ghaffar Alsharifa^39^, Taher AlMahroos^39^, Wasan A. M. Al Taie^40^, Walaa.K.E.Mohamed^41^, Hussaina Banu^42^, Naiema Almhdi Ocab^43^, Amal Sharif Eljali^44^, Ehsan Elmahdi^45^, Hamed Sami.^46^

^1^Faculty of Medicine, Aleppo University, Aleppo, Syria

^2^Faculty of Medical Science, Lebanese University, Hadath, Lebanon

^3^Faculty of Medicine, Cairo University, Cairo, Egypt

^4^Polydisciplinary Faculty, Sultan Moulay Slimane University, Beni Mellal, Morocco

^5^Biology Department, Biology Engineering Laboratory, Functional and Pathological Biology Team, Faculty of Sciences and Techniques, Sultan Moulay Slimane University, Beni Mellal. Morocco

^6^Faculty of Sciences and Techniques, Sidi Mohammed Ben Abdellah University, Fez, Morocco

^7^Faculty of Science, Damanhour University, Beheira, Egypt

^8^Faculty of Medicine, Alexandria University, Alexandria, Egypt

^9^Faculty of Pharmacy, Modern University for Technology and Information, Cairo, Egypt

^10^Ministry of Health, Alexandria, Egypt

^11^Faculty of Agriculture Biotechnology Department, Benha University, Benha, Egypt

^12^Faculty of Medicine, University of Abou Bekr Belkaid, Tlemcen, Algeria

^13^Faculty of Medicine, University of Oran Ahmed Ben Bella 1, Oran, Algeria

^14^Faculty of Medicine, University of Tlemcen, Tlemcen, Algeria

^15^Faculty of Dental Medicine, Lebanese University, Hadath, Lebanon

^16^Faculty of Medicine, Beirut Arab University, Beirut, Lebanon

^17^Faculty of Medicine, Al-Quds University, Gaza, Palestine

^18^Department of Medical Laboratory Sciences, Hebron University, Hebron, Palestine

^19^Faculty of Medicine, Al-Azhar University-Gaza, Gaza, Palestine

^20^Faculty of Medicine, Alquds University, Jerusalem, Palestine

^21^Faculty of Medicine, Khartoum University, Khartoum, Sudan

^22^Faculty of Medicine, University of Gezira, Wad Madani, Sudan

^23^Faculty of Medicine, Gezira College of Medical Sciences and Technology, Wed Madani, Sudan

^24^Faculty of Medicine, Hashemite University, Zarqa, Jordan

^25^Department of Internal Medicine, King Fahad Specialist Hospital, Tabuk, Saudi Arabia

^26^Faculty of Pharmacy, Alexandria University, Alexandria, Egypt

^27^Faculty of Medicine, Kuwait University, Kuwait City, Kuwait

^28^Department of Suegery, Jaber AlAhmad AlSabah Hospital, Kuwait City, Kuwait

^29^Department of Emergency, Rania Teaching Hospital, Rania, Sulaymaniah, Kurdistan-region, Iraq

^30^College of Education for Woman, Tikrit University, Tikrit, Iraq

^31^Faculty of Medicine, Jordan University of Science and Technology, Irbid, Jordan

^32^Emergency department, Princess salma hospital, Amman, Jordan

^33^Faculty of Pharmacy, Hashemite University, Amman, Jordan

^34^Faculty of Medicine, Alzaiem Alazhari University, Khartoum, Sudan

^35^Faculty of Sport, Hodeidah University, Hodeidah, Yemen

^36^Faculty of Dentistry, Emirates International University, Sana'a, Yemen

^37^Faculty of Medicine and Health Sciences, Sana'a University, Sana'a, Yemen

^38^Faculty of Clinical Pharmacy, Hodeidah University, Hodeidah City, Yemen

^39^Faculty of Medicine, Arabian Gulf University, Manama, Bahrain

^40^RAK College of Dental Sciences, RAK Medical and Health Sciences University, Ras Al Khaimah, United Arab Emirates

^41^Department of Genetics and Microbiology, Autonomous University of Barcelona, Barcelona, Spain

^42^College of Health Sciences, Abu Dhabi University, Al Ain, United Arab Emirates

^43^Faculty of Pharmacy, Omar Al-Mukhtar University, Tobruk, Libya

^44^Faculty of Medicine, Tobruk University, Tobruk, Libya

^45^Faculty of Medicine, Benghazi University, Benghazi, Libya

^46^Faculty of Pharmacy, 6 October University, Giza, Egypt

## Author contributions

NE questionnaire design, web-survey design, supervised the data collection process, and checked writing. SN researched literature, questionnaire design, web survey design, coordinate and monitor the data collection process with collaborators, and wrote the first draft of the manuscript. RS interpret data, organizing and data arrangement, coded data, involved in statistical analysis, designed figures, and checked writing. RM manuscript editing. NH supervised all steps, checked writing, and approved methodology. The EARG Group collected the data. All authors have seen and approved the final version of the manuscript.

## Conflict of interest

The authors declare that the research was conducted in the absence of any commercial or financial relationships that could be construed as a potential conflict of interest.

## Publisher's note

All claims expressed in this article are solely those of the authors and do not necessarily represent those of their affiliated organizations, or those of the publisher, the editors and the reviewers. Any product that may be evaluated in this article, or claim that may be made by its manufacturer, is not guaranteed or endorsed by the publisher.
