# Supplementary material for: Knowledge, practice and attitude toward anabolic hormones and nutritional supplements among people practicing sports in the MENA region before and during COVID-19 lockdown
Source: Front Public Health. 2022 Oct 17;10:1018757. doi: 10.3389/fpubh.2022.1018757 (PMC9618939; doi:10.3389/fpubh.2022.1018757)
Supplement: Supplementary file 1 [file Table_1.DOCX]

**Table S1: Descriptive analysis for non-scored KAP questions:**

| Questions | **Answers** | **Count (%)** |
| --- | --- | --- |
| Knowledge | | |
| Who told you to use hormones and supplements? | **A doctor** | 123 (2.1%) |
|  | **A nutritionist** | 134 (2.3%) |
|  | **A pharmacist** | 44 (0.75%) |
|  | **A trainer** | 300 (5.1%) |
|  | **Internet** | 246 (4.2%) |
|  | **Myself** | 40 (0.68%) |
|  | **Others** | 273 (4.7%) |
| What is your source of information about hormones and supplements? | **A trainer** | 682 (11.7%) |
|  | **A doctor** | 1248 (21.4%) |
|  | **Friends** | 2290 (39.2%) |
|  | **Internet** | 3741 (64%) |
| Attitude | | |
| Why do you use hormones and supplements? | **Body building** | 480 (8.2%) |
|  | **Protection from disease** | 172(2.9%) |
|  | **Improve performance** | 355(6.1%) |
|  | **Weight loss** | 143(2.4%) |
| Do you think supplements and hormones help you with any of the following? | **Win championships** | 1847(31.6%) |
|  | **Look Better** | 3874(66.3%) |
|  | **Make athlete and strong** | 5845(100%) |
| Practice |  |  |
| What type of supplements you use? [Before COVID-19 lockdown] | **Proteins** | 837 (14.3%) |
|  | **Energy bar (carbohydrate)** | 77 (1.3%) |
|  | **Vitamins** | 401 (6.9%) |
|  | **Sport drinks** | 62 (1.1%) |
| What type of supplements you use? [During COVID-19 lockdown] | **Proteins** | 405 (6.9%) |
|  | **Energy bar (carbohydrate)** | 71 (1.2%) |
|  | **Vitamins** | 422 (7.2%) |
|  | **Sport drinks** | 52 (0.9%) |
| What type of hormones you use? [Before COVID-19 lockdown] | **Anabolic steroids** | 380 (6.5%) |
|  | **insulin** | 27 (0.5%) |
|  | **Growth Hormone (GH)** | 44 (0.75%) |
|  | **Cortisol** | 24 (0.4%) |
| What type of hormones you use? [During COVID-19 lockdown] | **Anabolic steroids** | 241 (4.1%) |
|  | **insulin** | 22 (0.4%) |
|  | **Growth Hormone (GH)** | 28 (0.5%) |
|  | **Cortisol** | 6 (0.1%) |
| What is your route of administration to these hormones? | **Injection** | 71 (1.2%) |
|  | **Tablets** | 93 (1.6%) |
|  | **Both** | 83 (1.4%) |
|  | **Don't use** | 475 (8.1%) |
| From where you get hormones and supplements? [Before COVID-19 lockdown] | **Gym Trainer** | 188 (3.2%) |
|  | **Online** | 199 (3.4%) |
|  | **Pharmacy** | 335 (5.7%) |
| From where you get hormones and supplements? [During COVID-19 lockdown] | **Gym Trainer** | 169 (2.9%) |
|  | **Online** | 229 (3.9%) |
|  | **Pharmacy** | 324 (5.5%) |
| Have you had any of these problems when you stopped using hormones? | **Fluctuations in mood and depression** | 95 (1.6%) |
|  | **Anorexia** | 47 (0.8%) |
|  | **Anxiety and insomnia** | 63 (1.1%) |
|  | **Decrease in Fitness** | 53 (0.9%) |
|  | **Muscle weakness** | 102 (1.8%) |
|  | **Desire to return to hormones again** | 56 (1%) |
|  | **I Don't Have Any Symptoms** | 90 (1.5%) |
|  | **I Don't Use Hormones** | 441 (7.6%) |
|  | **I did not stop using** | 39 (0.7%) |
