# Supplementary material for: Knowledge, practice and attitude toward anabolic hormones and nutritional supplements among people practicing sports in the MENA region before and during COVID-19 lockdown
Source: Front Public Health. 2022 Oct 17;10:1018757. doi: 10.3389/fpubh.2022.1018757 (PMC9618939; doi:10.3389/fpubh.2022.1018757)
Supplement: Supplementary file 2 [file Table_2.DOCX]

**Table S2: Comparative analysis between types of supplements used before and during COVID-19 lockdown: (Practice)**

|  | Before COVID-19 era | During COVID-19 era | P value | McNemar's X^2^ |
| --- | --- | --- | --- | --- |
| Proteins | 837 (14.3%) | 405 (6.9%) | **<0.001 ***** | **3912.5** |
| Energy bar (carbohydrate) | 77 (1.3%) | 71 (1.2%) | **<0.001 ***** | **5556.5** |
| Vitamins | 401 (6.9%) | 422 (7.2%) | **<0.001 ***** | **4297.7** |
| Sport drinks | 62 (1.1%) | 52 (0.9%) | **<0.001 ***** | **5626.9** |
