# Supplementary material for: Knowledge, practice and attitude toward anabolic hormones and nutritional supplements among people practicing sports in the MENA region before and during COVID-19 lockdown
Source: Front Public Health. 2022 Oct 17;10:1018757. doi: 10.3389/fpubh.2022.1018757 (PMC9618939; doi:10.3389/fpubh.2022.1018757)
Supplement: Supplementary file 3 [file Table_3.DOCX]

**Table S3: Comparative analysis between types of hormones used before and during COVID-19 lockdown: (Practice)**

|  | Before COVID-19 lockdown | During COVID-19 lockdown | P value | McNemar's X2 |
| --- | --- | --- | --- | --- |
| Anabolic steroids | 380 (6.5%) | 241 (4.1%) | **<0.001 ***** | **4780.9** |
| Insulin | 27 (0.5%) | 22 (0.4%) | **<0.001 ***** | **5750.3** |
| Growth Hormone (GH) | 44 (0.75%) | 28 (0.5%) | **<0.001 ***** | **5715.6** |
| Cortisol | 24 (0.4%) | 6 (0.1%) | **<0.001 ***** | **5801** |
