# Supplementary material for: Knowledge, practice and attitude toward anabolic hormones and nutritional supplements among people practicing sports in the MENA region before and during COVID-19 lockdown
Source: Front Public Health. 2022 Oct 17;10:1018757. doi: 10.3389/fpubh.2022.1018757 (PMC9618939; doi:10.3389/fpubh.2022.1018757)
Supplement: Supplementary file 4 [file Table_4.DOCX]

**Table S4: Comparative analysis between sources of getting hormones and supplements before and during COVID-19 lcokdown: (Practice)**

|  | Before COVID-19 era | During COVID-19 era | P value | McNemar's X2 |
| --- | --- | --- | --- | --- |
| Gym Trainer | 188 (3.2%) | 169 (2.9%) | **<0.001 ***** | **5167.7** |
| Online | 199 (3.4%) | 229 (3.9%) | **<0.001 ***** | **4992.9** |
| Pharmacy | 335 (5.7%) | 324 (5.5%) | **<0.001 ***** | **4608.2** |
